# Augmenting Mortality Prediction in Critically Ill Adults With Medication Data and Machine Learning Models

**DOI:** 10.1097/CCE.0000000000001331

**Published:** 2025-10-07

**Authors:** Brian Murray, Tianyi Zhang, Zhetao Chen, Xianyan Chen, Bokai Zhao, Susan E. Smith, John W. Devlin, David J. Murphy, Rishikesan Kamaleswaran, Andrea Sikora

**Affiliations:** 1 Department of Clinical Pharmacy, University of Colorado Skaggs School of Pharmacy, Aurora, CO.; 2 Department of Statistics, University of Georgia Franklin College of Arts and Sciences, Athens, GA.; 3 Department of Epidemiology and Biostatistics, University of Georgia College of Public Health, Athens, GA.; 4 Department of Clinical and Administrative Pharmacy, University of Georgia College of Pharmacy, Athens, GA.; 5 Department of Pharmacy and Health Systems Sciences, Bouve College of Health Sciences, Northeastern University, Boston, MA.; 6 Division of Pulmonary and Critical Care Medicine, Brigham and Women’s Hospital, Boston, MA.; 7 Department of Pulmonary, Allergy, Critical Care and Sleep Medicine, Emory University School of Medicine, Atlanta, GA.; 8 Department of Surgery, Duke University School of Medicine, Durham, NC.; 9 Department of Biomedical Informatics, University of Colorado School of Medicine, Aurora, CO.

**Keywords:** artificial intelligence, critical care, machine learning, medication regimen complexity, mortality

## Abstract

**BACKGROUND:**

Mortality prediction in ICU adults is only marginally improved when medication regimen complexity (MRC) data is incorporated into traditional regression models. Machine learning (ML) may improve this prediction.

**OBJECTIVE:**

To compare the performance of different ML approaches incorporating MRC data to both traditional and advanced regression approaches, with and without MRC data, to predict hospital mortality in ICU adults.

**DERIVATION COHORT:**

Nine hundred ninety-one ICU adults at the University of North Carolina (UNC) Health System.

**VALIDATION COHORT:**

A temporally distinct cohort of 4,878 ICU adults at UNC and an external cohort of 12,290 ICU adults at the Oregon Health and Science University.

**PREDICTION MODEL:**

Supervised, classification-based ML models (e.g., Random Forest, Support Vector Machine [SVM], and XGBoost) were developed. Twenty-seven variables at ICU baseline (age, sex, service, diagnosis) and 24 hours (illness severity, supportive care use, fluid balance, laboratory values, MRC-ICU, vasopressor use) associated with mortality, and 14 missingness indicator variables, were included in each ML model. Traditional and advanced (equipped with linear predictors, predictors in nature cubic splines, predictors in smoothing cubic splines, and local linear predictors) regression models were optimized using stepwise selection by Bayesian Information Criterion. Area under the receiver operating characteristic (AUROC) was compared among models.

**RESULTS:**

Random Forest, SVM, and XGBoost achieved AUROCs of 0.83, 0.85, and 0.82, respectively, on the test set. Traditional regression models based on Sequential Organ Failure Assessment, Acute Physiology and Chronic Health Evaluation (APACHE) II, MRC-ICU + Sequential Organ Failure Assessment + APACHE II with and without an interaction term, and a full model including all 27 available variables demonstrated AUROCs of 0.81, 0.72, 0.82, 0.83, and 0.86, respectively. Advanced regression models yielded AUROCs of 0.85, 0.86, 0.85, and 0.84, respectively. The MRC-ICU exhibited a moderate level of feature importance in both XGBoost and Random Forest models. Models demonstrated lower performance in the validation cohorts.

**CONCLUSIONS:**

Use of ML, compared with traditional and advanced regression methods, did not improve hospital mortality prediction despite medication data inclusion. The MRC-ICU demonstrates moderate feature importance in select ML models.

**KEY POINTS**

**Question:** Can machine learning (ML) methods incorporating medication data improve hospital mortality prediction for ICU adults compared with traditional or advanced regression models?

**Findings:** ML methods incorporating medication data did not improve hospital mortality prediction compared with traditional or advanced regression models. Although medications were features of moderate importance in select ML models, they were not important in the advanced regression models.

**Meaning:** Although ML methods may be beneficial for uncovering complex relationships, simpler and more transparent regression methods appear to achieve comparable performance to ML methods for mortality prediction in ICU adults.

Mortality prediction for adults admitted to the ICU is important for clinical decision-making, outcomes research, and quality improvement (1, 2). Therefore, improving the accuracy of ICU mortality prediction models remains an important ongoing area of research (3–5). Severity of illness scores (e.g., Acute Physiology and Chronic Health Evaluation [APACHE), Sequential Organ Failure Assessment [SOFA]) are commonly used to predict mortality in the ICU but have limitations (6–9). Although these scores are reflective of syndrome-attributable mortality, neither considers the impact of daily ICU patient management.

Although medications are key influencers of mortality (10–14), they are rarely included in ICU mortality prediction models (15–21). Given the causal role of medications on outcomes in critically ill adults, incorporating this information into mortality prediction models may improve model performance and better inform ICU care (22).

The addition of the medication regimen complexity in the ICU (MRC-ICU) score to regression models that included both APACHE II and SOFA only modestly improved mortality prediction, and every 1-point increase in the MRC-ICU score was unexpectedly found to be associated with a 6% reduction in mortality (23). However, in this study, the time-dependency of factors affecting mortality was not considered (i.e., only baseline rather than daily ICU variables were included), and the results were not validated in an independent dataset (24). Realizing the inter-relationship between severity of illness, the MRC-ICU score, and mortality is complicated and potentially nonlinear, models capable of managing such complexity may also improve hospital mortality prediction in ICU adults.

Machine learning (ML) approaches can process vast amounts of information and identify previously unelucidated patterns in data, potentially improving prediction models through the incorporation of a greater number of latent outcome determinants and markers with predictive value. Using ML, it is possible to incorporate specific and granular medication data with severity of illness variables to create more comprehensive outcome prediction models. ML models incorporating medication data have demonstrated improved prediction of fluid overload and duration of mechanical ventilation in critically ill patients but have not been rigorously used as a method to predict mortality (25, 26).

The primary objective of this study was to compare the performance of ML models for hospital mortality prediction that considered medication data (as summarized in the MRC-ICU score), a broad range of clinical variables, and severity of illness scores (APACHE II, SOFA) with traditional and advanced regression models both including and not including the MRC-ICU score. We hypothesized that ML modeling approaches would demonstrate superior mortality prediction compared with either traditional or advanced regression models and that medication data would be highly ranked in feature importance graphs for ML models.

## MATERIALS AND METHODS

This retrospective, observational cohort study was reviewed by the institutional review boards (IRBs) at the University of Georgia (UGA), the University of North Carolina (UNC), and Oregon Health and Science University (OHSU) and deemed to be exempt from IRB oversight (IRB Waiver Number Project 00001541). All methods were performed in accordance with the ethical standards of the UGA IRB and the Helsinki Declaration of 1975. This evaluation followed the Strengthening the Reporting of Observational Studies in Epidemiology (STROBE) and Transparency Reporting of a multivariable prediction model for Individual Prognosis Or Diagnosis-Artificial Intelligence (TRIPOD–AI) extension reporting frameworks, as applicable **(Supplemental Digital Content**, **Appendices 1** and **2**, https://links.lww.com/CCX/B560) (27, 28).

### Dataset Development

Patient data were obtained from the UNC Health System, an integrated healthcare delivery system, via the Carolina Data Warehouse, which houses EPIC electronic health record (EHR) data, and from OHSU via the Oregon Clinical and Translational Research Institute data warehouse.

Given the intensity of the data collection effort, random number generation was used to identify a sample of 1,000 adults aged 18 years old or older admitted to the ICU for greater than or equal to 24 hours between October 2015 and October 2020 (UNC 1000 dataset) to serve as the training/test set. Patients were excluded if data were not from their index ICU admission or if they were placed on comfort care within the first 24 hours of their ICU stay. Detailed information on this cohort has been previously published (25, 29). To evaluate all ML and regression models, the UNC 1000 cohort was split into training and test sets, using a ratio of 4:1. Two datasets were used for validation: 1) a temporally distinct (January 2021–December 2023) random sample of 5,000 ICU adult patients from the same health system (UNC 5000 dataset) and 2) a cohort of 12,290 ICU patients from OHSU (OHSU dataset).

### Data Collection, Outcomes, and Covariates

The primary outcome was hospital mortality. The EHR was queried to obtain data for 27 variables previously reported to influence hospital mortality among adults admitted to the ICU (12, 30–35). The four ICU baseline characteristics included age, sex, type of admission ICU (i.e., burn, cardiovascular, cardiothoracic, medical, neurosciences, surgical, mixed), and primary ICU admission diagnosis (i.e., burn, cardiovascular, dermatology, electrolyte abnormalities, endocrine, fever, gastrointestinal, hematologic, hepatic, infection, mental health, neoplasm, neurology, pneumonia, pregnancy, pulmonary, renal, respiratory, sepsis, shock, syncope, toxicology/ingestion, trauma, weakness, or other). The 21 ICU clinical variables (collected 24 hr after ICU admission) included APACHE II and SOFA score (using worst values in this 24 hr period), vital signs [i.e., heart rate, hypotension (systolic blood pressure < 90 mm Hg), temperature], acute respiratory distress syndrome (ARDS) classification (i.e., mild, moderate, or severe based on ratio of partial pressure of arterial oxygen (Pao_2_) to Fio_2_ [Fio_2_]), use of supportive care devices (i.e., continuous renal replacement therapy, mechanical ventilation), serum laboratory values (i.e., albumin, bicarbonate, creatinine, glucose, lactate, potassium, pH, sodium, hemoglobin, hematocrit, platelets, WBC count), and fluid balance (L). The two medication variables, also collected 24 hours after ICU admission, included the MRC-ICU score and vasopressor use.

### Data Analysis

Any missing SOFA or APACHE II score component was assumed to be normal (**Supplemental Table 1**, https://links.lww.com/CCX/B560). Dummy Variable Adjustment for missingness was conducted for 14 predictors (i.e., heart rate, systolic blood pressure, temperature, ARDS classification, albumin, bicarbonate/creatinine/sodium, glucose, hemoglobin/hematocrit, lactate, pH, platelets, potassium, WBC count, and fluid balance at 24 hr) to estimate how much the missing cases differed from an average individual without missing data for the primary outcome of mortality (36).

#### Machine Learning Analysis

Three ML models, Random Forest, Support Vector Machine (SVM), and XGBoost, were developed using all collected variables described earlier (37–42). Feature importance graphs were used to visualize the strength of every predictor. Feature importance was measured by the mean decrease in node impurity, the absolute magnitude of the coefficients for each variable with a normalized dataset, and the frequency of a feature’s usage in the trees for Random Forest, SVM, and XG Boost models, respectively.

#### Traditional Regression Analysis

Based on a prior study (23), a traditional logistic regression model including SOFA, APACHE II, and the MRC-ICU score as predictors was constructed and benchmarked against models including only SOFA or APACHE II as predictor variables. Given the face validity of collected variables and potential impact on mortality, a linear regression model was constructed using all 27 variables (full model). Due to potential interactions between SOFA, APACHE II, and MRC-ICU, a regression model including an interaction term was constructed.

#### Advanced Regression Analysis

Four advanced regression models (i.e., linear predictors, predictors in nature cubic splines, predictors in smoothing cubic splines, and local linear predictors) were each constructed with the 27 variables reported above along with an additional 14 indicators for missingness (**Supplemental Table 2**, https://links.lww.com/CCX/B560). Stepwise variable selection with Bayesian Information Criterion was used to select the final models (43).

To assess both the specific and overall impact of each predictor in the regression models, we conducted two types of regression analyses: 1) individual logistic regressions examining a single variable alongside its relevant missingness indicator (if applicable), and 2) a comprehensive additive logistic regression encompassing all predictors and their respective missingness indicators. During model training, five-fold cross-validation was applied to choose the hyperparameters for local linear logistic regression and the three ML models. For local linear logistic regression, the proportion of observations in a neighborhood of every point to be used for fitting the models was tuned. For Random Forest, two hyperparameters were tuned (number of trees and number of variables randomly sampled as candidates at each split). For SVM, linear kernel was used, and cost of constraints violation was tuned. For XGBoost, two hyperparameters were tuned (maximum depth of a tree and maximum number of boosting iterations). The models were fitted on the training set after the optimal hyperparameters were selected. Then, predictions for mortality for all 10 models were made using the test set.

To evaluate the predictive abilities of each model on hospital mortality, area under the receiver operating characteristic curve (AUROC) was computed in addition to sensitivity, specificity, negative predictive value (NPV), positive predictive value (PPV) (or precision), and accuracy in the test set. Model calibrations were determined, and model AUROCs were compared using DeLong’s test where prediction thresholds were chosen by maximizing Informedness, Matthew’s Correlation Coefficient (MCC) and F1 scores in the training set (44). A two-sided *p* value of less than 0.05 was used to determine statistical significance for all variables. All analyses were performed using R (Version 4.3.0; R Foundation for Statistical Computing, Vienna, Austria).

### Validation

Model performance was further validated using the UNC 5000 and OHSU datasets. This process involved applying trained models to the validation data, generating ROC curves, and assessing model metrics using the same processes as described above.

## RESULTS

### Training/Test Set

From the 1,000 patients randomly selected for inclusion in the training/test sets, 9 were excluded because the ICU admission in the dataset did not represent their index ICU admission, leaving 991 patients in the final cohort. Patients were 56.8% male, had an average age of 61.2 (sd 17.6) years, and were predominantly admitted to medical (40.8%) or cardiac/cardiothoracic (30.9%) ICUs, with cardiovascular (25.5%), acute respiratory (12.5%), or neurologic (12.2%) conditions representing the most common ICU admission diagnosis categories. A total of 97 (9.8%) of the patients died during their ICU hospitalization. Abbreviated demographic characteristics for the test/training cohort are described in **Table 1** (full demographics in **Supplemental Table 3**, https://links.lww.com/CCX/B560).

**TABLE 1.** Abbreviated Comparison of Model Variables in the Training/Test Cohort for Patients Who Died in the Hospital vs. Those Who Did Not^a^

Variable	All (*n* = 991)	Mortality (*n* = 97)	No Mortality (*n* = 894)	*p*
Age, mean (sd)	61.2 (17.6)	66.8 (15.5)	60.6 (17.7)	< 0.01
Male sex, *n* (%)	563 (56.8)	56 (57.7)	507 (56.7)	0.93
ICU type, *n* (%)				0.01
Medical ICU	404 (40.8)	53 (54.6)	351 (36.3)	
Cardiac/cardiothoracic ICU	306 (30.9)	20 (20.6)	286 (32.0)	
Surgical ICU	97 (9.8)	4 (4.1)	93 (10.4)	
Other	184 (18.6)	20 (20.6)	164 (18.3)	
Primary ICU admission diagnosis, *n* (%)				< 0.01
Cardiovascular	253 (25.5)	11 (11.3)	242 (27.0)	
Acute respiratory	124 (12.5)	25 (25.8)	99 (11.1)	
Neurologic	121 (12.2)	12 (12.4)	109 (12.2)	
Sepsis	107 (10.8)	17 (17.5)	90 (10.1)	
Acute gastrointestinal/hepatic	83 (8.4)	8 (8.2)	75 (8.4)	
Other	303 (30.6)	24 (24.7)	279 (31.2)	
24 hr after ICU admission, mean (sd) unless otherwise noted				
Acute Physiology and Chronic Health Evaluation II	14.1 (6.4)	20.6 (6.0)	13.4 (6.0)	< 0.01
Sequential Organ Failure Assessment	5.2 (4.2)	9.8 (3.9)	4.7 (3.9)	< 0.01
Heart rate	105.5 (21.6)	114.5 (25.0)	104.5 (21.0)	< 0.01
Hypotension (systolic blood pressure < 90 mm Hg), *n* (%)	316 (32.3)	47 (50.0)	269 (30.4)	< 0.01
Temperature (F)	98.5 (3.9)	98.3 (2.5)	98.5 (4.0)	0.55
Acute respiratory distress syndrome classification, *n* (%)				< 0.01
Mild	95 (25.8)	9 (12.5)	86 (29.0)	
Moderate	145 (39.3)	37 (51.4)	108 (36.4)	
Severe	46 (12.5)	14 (19.4)	32 (10.8)	
Continuous renal replacement therapy at 24 hr, *n* (%)	11 (1.1)	3 (3.1)	8 (0.9)	0.15
Mechanical ventilation at 24 hr, *n* (%)	291 (29.4)	55 (56.7)	236 (26.4)	< 0.01
Albumin mg/dL	2.9 (0.7)	2.5 (0.7)	3.0 (0.7)	< 0.01
Creatinine mg/dL	1.6 (2.0)	2.3 (2.1)	1.5 (2.0)	< 0.01
Lactate mmol/L	2.6 (2.5)	3.8 (3.6)	2.3 (2.0)	< 0.01
pH < 7.2, *n* (%)	46 (11.3)	18 (23.7)	28 (8.4)	< 0.01
pH > 7.5, *n* (%)	32 (7.8)	11 (14.5)	21 (6.3)	< 0.01
Hemoglobin, g/dL	10.9 (2.4)	9.8 (2.4)	11.1 (2.4)	< 0.01
WBCs ×10^3^/μL	11.8 (6.6)	14.8 (10.4)	11.5 (5.9)	< 0.01
Fluid balance at 24 hr (L)	0.7 (2.4)	0.9 (2.2)	0.7 (2.5)	0.43
Medication regimen complexity-ICU score	10.3 (7.7)	14.3 (8.3)	9.9 (7.5)	< 0.01
Vasopressor use at 24 hr, *n* (%)	231 (23.3)	46 (47.4)	185 (20.7)	< 0.01

a Table excludes patients with missing data.

### Feature Selection and Model Performance

**Figure 1.**
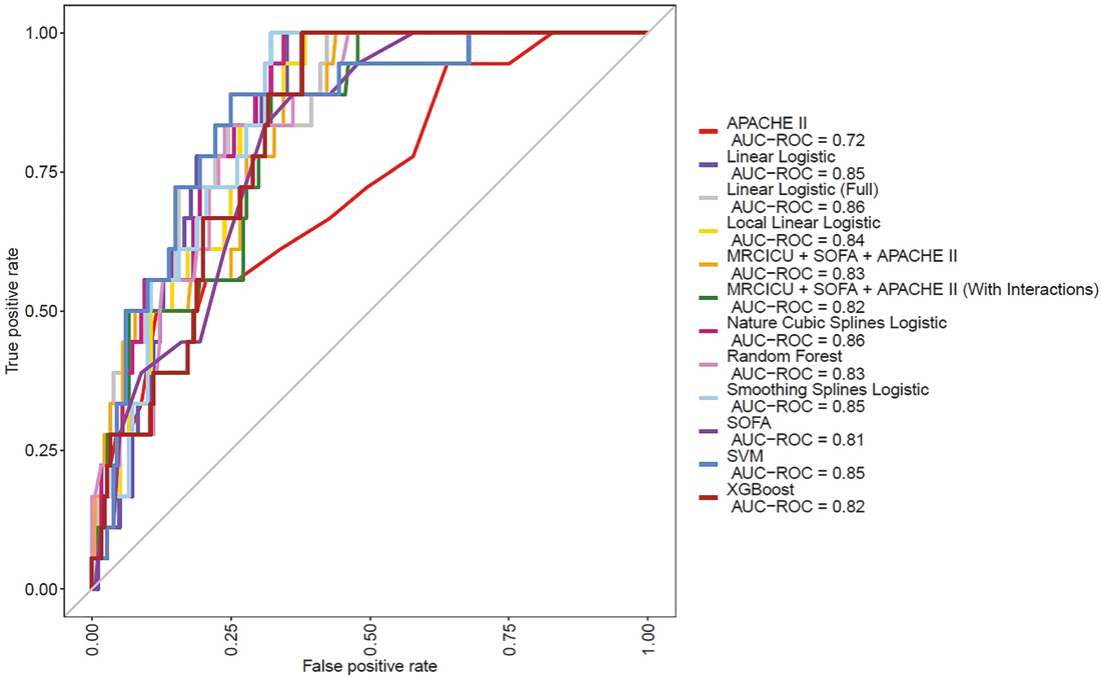
Area under the receiver operating characteristic curve (AUROC) for hospital mortality prediction on the test set. AUROCs for mortality prediction in critically ill adult patients admitted to the ICU were similar for machine learning-based and advanced regression models compared with models based on traditional regression methods and standard severity of illness metrics. APACHE II = Acue Physiology and Chronic Health Evaluation II, MRC-ICU = medication regimen complexity-ICU, SOFA = Sequential Organ Failure Assessment, SVM = Support Vector Machine.

On the testing dataset, ML models based on Random Forest, SVM, and XGBoost achieved AUROCs of 0.83, 0.85, and 0.82, respectively. Traditional logistic regression models based on APACHE II, SOFA, MRC-ICU+SOFA+APACHE II with and without an interaction term, and a full model including all 27 collected variables demonstrated AUROCs of 0.72, 0.81, 0.82, 0.83, and 0.86, respectively. Advanced models with stepwise variable selection procedure using logistic regression, nature cubic splines, smoothing cubic splines, and local linear logistic, yielded AUROCs of 0.85, 0.86, 0.85, and 0.84, respectively. Differences in model performance did not reach statistical significance. AUROC curves for all 12 models are shown in **Figure 1**; AUROCs and their corresponding CIs for each model are provided in **Supplemental Table 4** (https://links.lww.com/CCX/B560). By DeLong’s test, the AUROC for the best-performing ML model (SVM) was indistinguishable from the linear logistic model (*p* = 0.98); likewise, Random Forest vs. linear logistic showed no significant difference (*p* = 0.67). Detailed model performance metrics including accuracy, sensitivity, specificity, PPV, and NPV values on the test set with thresholds chosen by different criteria are reported in **Table 2**. The results of the univariate and multivariate analyses are presented in **Supplemental Table 5** (https://links.lww.com/CCX/B560), and the results of the stepwise selection are presented in **Supplemental Table 6** (https://links.lww.com/CCX/B560).

**TABLE 2.** Accuracy, Sensitivity, Specificity, Negative Predictive Value, and Positive Predictive Value for Mortality Prediction Models on Test Set

Model	Accuracy	Sensitivity	Specificity	Positive Predictive Value	Negative Predictive Value
Maximizing Informedness					
APACHE II	0.77, 0.71–0.83	0.56, 0.34–0.75	0.79, 0.73–0.85	0.21, 0.12–0.35	0.95, 0.90–0.97
SOFA	0.66, 0.59–0.73	0.89, 0.67–0.97	0.64, 0.57–0.71	0.20, 0.13–0.30	0.98, 0.94–1.00
MRC-ICU + SOFA + APACHE II	0.75, 0.68–0.81	0.56, 0.34–0.75	0.77, 0.70–0.82	0.19, 0.11–0.32	0.95, 0.90–0.97
MRC-ICU + SOFA + APACHE II (with interactions)	0.72, 0.65–0.78	0.56, 0.34–0.75	0.74, 0.67–0.8	0.18, 0.1–0.29	0.94, 0.89–0.97
Linear logistic	0.81, 0.75–0.86	0.67, 0.44–0.84	0.83, 0.77–0.88	0.28, 0.17–0.43	0.96, 0.92–0.98
Linear logistic (full)	0.80, 0.74–0.85	0.72, 0.49–0.88	0.81, 0.74–0.86	0.27, 0.17–0.41	0.97, 0.92–0.99
Nature cubic splines logistic	0.82, 0.76–0.87	0.56, 0.34–0.75	0.84, 0.78–0.89	0.26, 0.15–0.42	0.95, 0.90–0.97
Smoothing splines logistic	0.81, 0.75–0.86	0.61, 0.39–0.80	0.83, 0.77–0.88	0.27, 0.16–0.42	0.96, 0.91–0.98
Local linear logistic	0.80, 0.74–0.86	0.56, 0.34–0.75	0.83, 0.77–0.88	0.24, 0.14–0.39	0.95, 0.90–0.97
Random Forest	0.71, 0.64–0.77	0.83, 0.61–0.94	0.69, 0.62–0.76	0.21, 0.13–0.32	0.98, 0.93–0.99
SVM	0.73, 0.66–0.79	0.89, 0.67–0.97	0.71, 0.64–0.77	0.24, 0.15–0.35	0.98, 0.95–1.00
XGBoost	0.81, 0.75–0.86	0.39, 0.20–0.61	0.85, 0.79–0.89	0.21, 0.10–0.37	0.93, 0.88–0.96
Maximizing Matthew’s correlation coefficient					
APACHE II	0.77, 0.71–0.83	0.56, 0.34–0.75	0.79, 0.73–0.85	0.21, 0.12–0.35	0.95, 0.90–0.97
SOFA	0.80, 0.74–0.86	0.44, 0.25–0.66	0.84, 0.78–0.89	0.22, 0.11–0.37	0.94, 0.89–0.97
MRC-ICU + SOFA + APACHE II	0.75, 0.69–0.81	0.56, 0.34–0.75	0.77, 0.71–0.83	0.20, 0.11–0.32	0.95, 0.90–0.97
MRC-ICU + SOFA + APACHE II (with interactions)	0.78, 0.72–0.84	0.56, 0.34–0.75	0.81, 0.74–0.86	0.22, 0.13––0.36	0.95, 0.9–0.97
Linear logistic	0.84, 0.78–0.89	0.50, 0.29–0.71	0.87, 0.82–0.91	0.28, 0.16–0.45	0.95, 0.90–0.97
Linear logistic (full)	0.85, 0.80–0.90	0.56, 0.34–0.75	0.88, 0.83–0.92	0.32, 0.19–0.50	0.95, 0.91–0.98
Nature cubic splines logistic	0.82, 0.76–0.87	0.56, 0.34–0.75	0.84, 0.78–0.89	0.26, 0.15–0.42	0.95, 0.90–0.97
Smoothing splines logistic	0.85, 0.80–0.90	0.33, 0.16–0.56	0.91, 0.85–0.94	0.26, 0.13–0.46	0.93, 0.88–0.96
Local linear logistic	0.85, 0.79–0.90	0.33, 0.16–0.56	0.90, 0.85–0.94	0.25, 0.12–0.45	0.93, 0.88–0.96
Random Forest	0.78, 0.72–0.84	0.72, 0.49–0.88	0.79, 0.72–0.84	0.25, 0.16–0.39	0.97, 0.92–0.99
SVM	0.83, 0.77–0.88	0.61, 0.39–0.80	0.85, 0.79–0.89	0.29, 0.17–0.45	0.96, 0.91–0.98
XGBoost	0.86, 0.80–0.90	0.28, 0.12–0.51	0.92, 0.87–0.95	0.25, 0.11–0.47	0.93, 0.88–0.96
Maximizing F1					
APACHE II	0.77, 0.71–0.83	0.56, 0.34–0.75	0.79, 0.73–0.85	0.21, 0.12–0.35	0.95, 0.90–0.97
SOFA	0.80, 0.74–0.86	0.44, 0.25–0.66	0.84, 0.78–0.89	0.22, 0.11–0.37	0.94, 0.89–0.97
MRC-ICU + SOFA + APACHE II	0.78, 0.72–0.84	0.56, 0.34–0.75	0.81, 0.74–0.86	0.22, 0.13–0.36	0.95, 0.90–0.97
MRC-ICU + SOFA + APACHE II (with interactions)	0.81, 0.75–0.86	0.50, 0.29–0.71	0.84, 0.78–0.89	0.24, 0.13–0.39	0.94, 0.90–0.97
Linear logistic	0.84, 0.79–0.89	0.44, 0.25–0.66	0.88, 0.83–0.92	0.28, 0.15–0.46	0.94, 0.89–0.97
Linear logistic (full)	0.85, 0.80–0.90	0.56, 0.34–0.75	0.88, 0.83–0.92	0.32, 0.19–0.50	0.95, 0.91–0.98
Nature cubic splines logistic	0.83, 0.77–0.88	0.56, 0.34–0.75	0.86, 0.80–0.90	0.28, 0.16–0.44	0.95, 0.91–0.97
Smoothing splines logistic	0.85, 0.80–0.90	0.33, 0.16–0.56	0.91, 0.85–0.94	0.26, 0.13–0.46	0.93, 0.88–0.96
Local linear logistic	0.85, 0.79–0.90	0.33, 0.16–0.56	0.90, 0.85–0.94	0.25, 0.12–0.45	0.93, 0.88–0.96
Random Forest	0.84, 0.79–0.89	0.28, 0.12–0.51	0.90, 0.85–0.94	0.22, 0.10–0.42	0.93, 0.88–0.96
SVM	0.83, 0.77–0.88	0.61, 0.39–0.80	0.85, 0.79–0.89	0.29, 0.17–0.45	0.96, 0.91–0.98
*XGBoost*	0.86, 0.80–0.90	0.28, 0.12–0.51	0.92, 0.87–0.95	0.25, 0.11–0.47	0.93, 0.88–0.96

APACHE II = Acue Physiology and Chronic Health Evaluation II, MCC = Matthew’s Correlation Coefficient, MRC-ICU = medication regimen complexity-ICU, SOFA = Sequential Organ Failure Assessment, SVM = Support Vector Machine.

**Figure 2.**
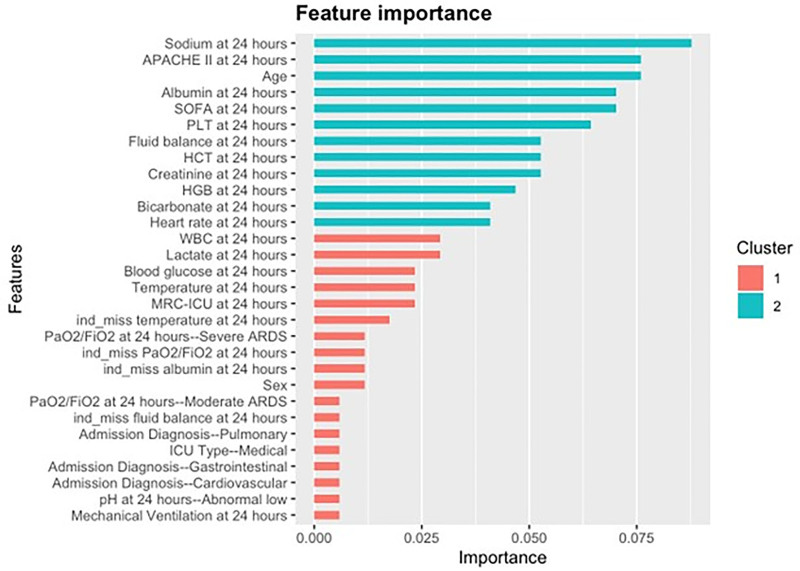
Feature importance for mortality prediction with XGBoost. Standard severity of illness metrics (Acue Physiology and Chronic Health Evaluation II [APACHE II], Sequential Organ Failure Assessment [SOFA]) demonstrated high feature importance in XGBoost ML-based models for mortality prediction in critically ill adult patients admitted to the ICU, as did other variables known to be associated with mortality in this population (e.g., age, fluid balance, etc.) Medication data as summarized in the medication regimen complexity-ICU (MRC-ICU) score demonstrated moderate feature importance similar to factors considered to have high clinical value for inpatient assessment (e.g., lactate, Pao_2_/Fio_2_ ratio). Machine learning-based modeling can identify and characterize complex relationships that may not be evident using traditional modeling techniques. ARDS = acute respiratory distress syndrome, HCT = hematocrit, HGB = hemoglobin, PLT = platelets.

Feature importance graphs for XGBoost and Random Forest models revealed similar contributing variables to the regression models (**Fig. 2**; and **Supplemental Fig. 1**, https://links.lww.com/CCX/B560); SOFA, APACHE II, age, heart rate, and key laboratory values were most prominently featured. Medication data in the form of the MRC-ICU score ranked as the seventeenth most important feature in both the XGBoost and Random Forest models, demonstrating moderate feature importance. Feature importance graphs generated for SVM models did not feature the MRC-ICU score **(Supplemental Fig. 2**, https://links.lww.com/CCX/B560).

### Validation

After excluding 122 patients from the temporally separate UNC5000 validation dataset due to their ICU admission not representing their initial ICU admission, validation was performed in 4,878 patients. Patients were 51.9% male and 58.9 (16.4) years old. Patients in this validation cohort were predominantly admitted to a medical ICU (94.9%) with acute respiratory (21.6%), sepsis (19.6%), or an acute gastrointestinal/hepatic issue (15.6%) as their primary ICU admission condition. A total of 964 (19.8%) of this validation cohort died during their ICU hospitalization. Full demographic characteristics for the UNC5000 validation cohort are described in **Supplemental Table 7** (https://links.lww.com/CCX/B560). Missingness characteristics for each variable are provided in **Supplemental Table 8** (https://links.lww.com/CCX/B560).

The AUROC curves for the prediction of hospital mortality when applying each trained model to the UNC5000 validation cohort are presented in **Supplemental Figure 3** (https://links.lww.com/CCX/B560). Model performance in this validation cohort was consistently lower than that in the test set across all models (**Supplemental Tables 9** and **10**, https://links.lww.com/CCX/B560). The ML models performed similarly to the traditional regression models and the advanced regression models. The Random Forest model exhibited the most stable discrimination from the test set to the validation set (AUROC 0.78) but had poor calibration.

The AUROC curves for the prediction of hospital mortality in the OHSU external validation set of 12,290 patients are presented in **Supplemental Figure 4** (https://links.lww.com/CCX/B560). Demographic characteristics and missingness for the external validation cohort are presented in **Supplemental Tables 11** and **12**, respectively (https://links.lww.com/CCX/B560). Model performance in the OHSU external validation cohort was consistently lower than that on the test set across all models; in particular, the full regression model demonstrated an AUROC of only 0.55 in the OHSU validation cohort compared with 0.86 in the test cohort **(Supplemental Tables 13** and **14**, https://links.lww.com/CCX/B560). The Random Forest model again exhibited the most stable discrimination from the test set to the external validation set with AUROC 0.80, but was also poorly calibrated.

Calibration curves for all models for the test set and validation sets are presented in **Supplemental Figures 5–7** (https://links.lww.com/CCX/B560).

## DISCUSSION

In our study, ML models did not outperform regression models for predicting hospital mortality, refuting our initial hypothesis. Compared with traditional and advanced regression, ML models identified different predictors of mortality and included the MRC-ICU score as a feature of moderate importance. The consistently lower AUROCs for each model on the validation sets may reflect dataset imbalances or model overfitting on the training set, highlighting the importance of external model validation as well as the potential value in retraining or fine-tuning models at an institutional level during clinical implementation to capture nuances of patient populations and patient care practices that may impact model performance. More stable performance of the Random Forest model in the validation sets may suggest an increased versatility or accuracy of some ML models over time or in different populations.

This study represents an important methodological advancement in our understanding of how to model medications as predictors of mortality in critical illness: this is the first multicenter validation study of mortality models to incorporate medication data that was benchmarked against standardized severity of illness scores with the goal to identify modeling approaches that are best able to handle the nonlinear relationships that we have previously observed (23, 25). The comparison of standard metrics, regression models, and supervised ML models within multiple datasets demonstrates important points for ML in the ICU. First, ML models performed similarly to regression models based only on severity of illness scores (i.e., APACHE II, SOFA) with or without the MRC-ICU score, highlighting the importance of ensuring any new prediction model compares favorably to established benchmarks. Second, this study demonstrates the ability of ML models to characterize complex relationships between variables and identify previously unknown associations that may not appear relevant with traditional regression, an important strength that may lead to improved outcome prediction and novel hypothesis generation with ML. Third, given ML models may only offer incremental performance benefits compared with simpler methods, barriers to ML adoption in the clinical setting must be considered (e.g., lack of explainability or interpretability [i.e., “black box” models] and implications on clinical acceptance, demands for information technology and model governance infrastructure, dataset bias, resource utilization, data quality and availability, ease of workflow integration). Fourth, models must be evaluated locally because performance on a training/test set does not ensure external performance (45). This principle was demonstrated in external validation of the Epic Sepsis Model, which after implementation in the clinical setting exhibited poor discrimination and calibration in predicting the onset of sepsis in external validation (46). Despite the strengths of ML, it is important to consider whether these complex models improve ICU outcomes prediction compared with regression models that are simpler to conduct and more transparent (25, 47).

Medications are important determinants of patient outcomes that may both treat disease and cause adverse events, exerting independent effects on mortality and other patient-centered outcomes (e.g., duration of mechanical ventilation, discharge disposition, post-intensive care syndrome). Including medications in prediction modeling and characterizing their associations with outcomes may not only improve our ability to predict outcomes, but our ability to intervene before an outcome occurs. Our previous work demonstrated that addition of the MRC-ICU score to conventional severity of illness scores (i.e., SOFA, APACHE II) only modestly improved hospital mortality prediction, but our approach had limitations and suggested the relationship between MRC-ICU and mortality is complex and nonlinear (23). Although the current study addresses many of the limitations of this prior work, it raises important questions about how ICU medication exposure affects ICU mortality. The models generated in our study each weighted the importance of medication data differently. Although medication exposure as estimated by the MRC-ICU score was not independently associated with hospital mortality in regression analyses, it was included as a feature of moderate importance in two of the ML models. In these two models, it had similar importance (i.e., association) to hospital mortality prediction as the serum lactate and fluid balance. These findings highlight the strengths of ML methods and the importance of further investigation (5, 16–20). The nonlinear relationship captured may be due to there being a “just right” amount of medication complexity for a given patient (e.g., broad-spectrum antibiotics and vasopressors may reduce mortality for a patient with septic shock, but the decision to add heavy sedation—which also increases the score—may increase complication risk).

Our study has several limitations. Methods in this evaluation are appropriate for prediction modeling but not causal inference; ML models identified the MRC-ICU score as a feature of moderate importance to hospital mortality prediction, but this statistical association does not provide causal or etiological information. The MRC-ICU score, although being associated with mortality (10–14), number and intensity of medication interventions (10, 48), need for mechanical ventilation (26), development of fluid overload (49), and the improvement of mortality prediction when added to conventional severity of illness scores (i.e., SOFA, APACHE II) (23), is not a comprehensive assessment of medication use and does not account for the effects of individual medications on mortality. APACHE II and SOFA were chosen for clinical benchmarking purposes, but they have notable limitations and newer tools (e.g., Global Open Source Severity of Illness Score [GOSSIS-1]) demonstrate superior mortality prediction (50); however, APACHE II and SOFA are valuable for clinical benchmarking due to ease of calculation, widespread clinical adoption, and clinician familiarity. Although a training/test set of 991 patients is large for ICU studies, it is likely underpowered for the purposes of ML modeling and this investigation. Inadequate sample sizes may result in model overfitting or homogenous/unrepresentative populations impacting generalizability, a potential contributor to the notably worse performance of most of the models in the validation cohorts compared with the test cohort and the poor calibration of most evaluated models in the test cohort. The size of the study cohorts and order of operations reflected the effort required to develop and process robust datasets including relevant medication data and the timing of cohort availability; future analysis would benefit from the use of larger, diverse and representative training cohorts. The management of missingness (i.e., single imputation with normal values for missing data) may alter model results and introduce bias; a multiple imputation approach has advantages and may be favored in some situations (51, 52). However, given considerations of computational efficiency, real-time handling of missingness when models are implemented in clinical settings, and missingness not at random for data points in critical care, single imputation with inclusion of missingness indicators is a reasonable approach that allows models to learn from patterns of missingness. Some continuous variables were dichotomized for clinical simplicity, potentially causing loss of information that impacted model performance (53); however, because data processing was identical for all models, this procedure did not affect model comparisons. Although we included a robust set of 27 variables reported to influence mortality, additional variables with predictive value may not have been collected; this includes variables after the first 24 hours of the ICU stay. Finally, the evaluation of hospital mortality (as opposed to 28-d or 90-d mortality), whereas the most feasible outcome to collect for this evaluation, may not be the optimal timepoint for outcome evaluation.

Our study has notable strengths. It represents the largest and most robust evaluation to date of the MRC-ICU score as well as the incorporation of MRC with severity of illness data and ML methods evaluating a patient-centered outcome that is relevant to potential future applications, with a training/test set of nearly 1,000 patients and two validation cohorts of approximately 5,000 and 12,000 patients, respectively. We included a robust set of demographic and clinical variables as potential predictors for model development. Our study also benchmarked developed models against traditional regression models based on classical severity of illness scores, a necessary metric for judging model performance in the critical care setting (54).

## CONCLUSION

Application of ML approaches including medication data as summarized in the MRC-ICU score did not improve mortality prediction compared with traditional regression approaches. Future studies should focus on application of ML approaches to larger cohorts with more granular and time-dependent medication data. Although ML approaches may improve prediction performance in some settings, performance should be benchmarked against simpler and more transparent models for outcome prediction.

## Supplementary Material
