# Supplementary material for: Augmenting Mortality Prediction in Critically Ill Adults With Medication Data and Machine Learning Models
Source: Crit Care Explor. 2025 Oct 7;7(10):e1331. doi: 10.1097/CCE.0000000000001331 (PMC12506993; doi:10.1097/CCE.0000000000001331)

Supplemental Digital Content

Table of Contents

Appendix 1 – STROBE Checklist 3

Appendix 2 – TRIPOD-AI Checklist 6

Supplemental Table 1 8

Supplemental Table 2 9

Supplemental Table 3 12

Supplemental Table 4 14

Supplemental Table 5 15

Supplemental Table 6 17

Supplemental Table 7 18

Supplemental Table 8 20

Supplemental Table 9 21

Supplemental Table 10 22

Supplemental Table 11 24

Supplemental Table 12 26

Supplemental Table 13 27

Supplemental Table 14 29

Supplemental Figure 1 30

Supplemental Figure 2 31

Supplemental Figure 3 32

Supplemental Figure 4 33

Supplemental Figure 5 34

Supplemental Figure 6 35

Supplemental Figure 7 36

	Item No.	Recommendation	Page No.
**Title and abstract**	1	(*a*) Indicate the study's design with a commonly used term in the title or the abstract	3
		(*b*) Provide in the abstract an informative and balanced summary of what was done and what was found	3
**Introduction**			
Background/rationale	2	Explain the scientific background and rationale for the investigation being reported	5-6
Objectives	3	State specific objectives, including any prespecified hypotheses	6
**Methods**			
Study design	4	Present key elements of study design early in the paper	7
Setting	5	Describe the setting, locations, and relevant dates, including periods of recruitment, exposure, follow-up, and data collection	7
Participants	6	*(b)* *Cohort study*—Give the eligibility criteria, and the sources and methods of selection of participants. Describe methods of follow-up<br>*Case-control study*—Give the eligibility criteria, and the sources and methods of case ascertainment and control selection. Give the rationale for the choice of cases and controls<br>*Cross-sectional study*—Give the eligibility criteria, and the sources and methods of selection of participants	7
		(*b*) *Cohort study*—For matched studies, give matching criteria and number of exposed and unexposed<br>*Case-control study*—For matched studies, give matching criteria and the number of controls per case	N/A
Variables	7	Clearly define all outcomes, exposures, predictors, potential confounders, and effect modifiers. Give diagnostic criteria, if applicable	7-8
Data sources/ measurement	8*	For each variable of interest, give sources of data and details of methods of assessment (measurement). Describe comparability of assessment methods if there is more than one group	7-8
Bias	9	Describe any efforts to address potential sources of bias	7-8
Study size	10	Explain how the study size was arrived at	7

Continued on next page

Quantitative variables	11	Explain how quantitative variables were handled in the analyses. If applicable, describe which groupings were chosen and why	7-10
Statistical methods	12	(*a*) Describe all statistical methods, including those used to control for confounding	8-10
		(*b*) Describe any methods used to examine subgroups and interactions	8-10
		© Explain how missing data were addressed	8
		(c) *Cohort study*—If applicable, explain how loss to follow-up was addressed<br>*Case-control study*—If applicable, explain how matching of cases and controls was addressed<br>*Cross-sectional study*—If applicable, describe analytical methods taking account of sampling strategy	N/A
		(*e*) Describe any sensitivity analyses	N/A
**Results**			
Participants	13*	(a) Report numbers of individuals at each stage of study—eg numbers potentially eligible, examined for eligibility, confirmed eligible, included in the study, completing follow-up, and analysed	11
		(b) Give reasons for non-participation at each stage	11
		(c) Consider use of a flow diagram	N/A
Descriptive data	14*	(a) Give characteristics of study participants (eg demographic, clinical, social) and information on exposures and potential confounders	11, Table 1
		(b) Indicate number of participants with missing data for each variable of interest	8, 9, Supplemental Content Table1, Supplemental Content Table 2
		(c) *Cohort study*—Summarise follow-up time (eg, average and total amount)	7
Outcome data	15*	*Cohort study*—Report numbers of outcome events or summary measures over time	11
		Case-control study—Report numbers in each exposure category, or summary measures of exposure	
		Cross-sectional study—Report numbers of outcome events or summary measures	
Main results	16	(*a*) Give unadjusted estimates and, if applicable, confounder-adjusted estimates and their precision (eg, 95% confidence interval). Make clear which confounders were adjusted for and why they were included	11-13, Figure 1, Supplemental Content
		(*b*) Report category boundaries when continuous variables were categorized	11-13, Figure 1, Supplemental Content
		(*c*) If relevant, consider translating estimates of relative risk into absolute risk for a meaningful time period	N/A

Continued on next page

Other analyses	17	Report other analyses done—eg analyses of subgroups and interactions, and sensitivity analyses	N/A
**Discussion**			
Key results	18	Summarise key results with reference to study objectives	14-16
Limitations	19	Discuss limitations of the study, taking into account sources of potential bias or imprecision. Discuss both direction and magnitude of any potential bias	15-16
Interpretation	20	Give a cautious overall interpretation of results considering objectives, limitations, multiplicity of analyses, results from similar studies, and other relevant evidence	14-16
Generalisability	21	Discuss the generalisability (external validity) of the study results	14-16
**Other information**			
Funding	22	Give the source of funding and the role of the funders for the present study and, if applicable, for the original study on which the present article is based	2

*Give information separately for cases and controls in case-control studies and, if applicable, for exposed and unexposed groups in cohort and cross-sectional studies.

Note: An Explanation and Elaboration article discusses each checklist item and gives methodological background and published examples of transparent reporting. The STROBE checklist is best used in conjunction with this article (freely available on the Web sites of PLoS Medicine at http://www.plosmedicine.org/, Annals of Internal Medicine at http://www.annals.org/, and Epidemiology at http://www.epidem.com/). Information on the STROBE Initiative is available at www.strobe-statement.org.

Section/Topic	Item	Development / evaluation[1]	Checklist item	Reported on page
**TITLE**				
Title	1	D;E	Identify the study as developing or evaluating the performance of a multivariable prediction model, the target population, and the outcome to be predicted	1
**ABSTRACT**				
Abstract	2	D;E	See TRIPOD+AI for Abstracts checklist	3
**INTRODUCTION**				
Background	3a	D;E	Explain the healthcare context (including whether diagnostic or prognostic) and rationale for developing or evaluating the prediction model, including references to existing models	5-6
	3b	D;E	Describe the target population and the intended purpose of the prediction model in the context of the care pathway, including its intended users (e.g., healthcare professionals, patients, public)	5-6
	3c	D;E	Describe any known health inequalities between sociodemographic groups	5-6
Objectives	4	D;E	Specify the study objectives, including whether the study describes the development or validation of a prediction model (or both)	6
**METHODS**				
Data	5a	D;E	Describe the sources of data separately for the development and evaluation datasets (e.g., randomised trial, cohort, routine care or registry data), the rationale for using these data, and representativeness of the data	7
	5b	D;E	Specify the dates of the collected participant data, including start and end of participant accrual; and, if applicable, end of follow-up	7
Participants	6a	D;E	Specify key elements of the study setting (e.g., primary care, secondary care, general population) including the number and location of centres	7
	6b	D;E	Describe the eligibility criteria for study participants	7
	6c	D;E	Give details of any treatments received, and how they were handled during model development or evaluation, if relevant	N/A
Data preparation	7	D;E	Describe any data pre-processing and quality checking, including whether this was similar across relevant sociodemographic groups	7-8
Outcome	8a	D;E	Clearly define the outcome that is being predicted and the time horizon, including how and when assessed, the rationale for choosing this outcome, and whether the method of outcome assessment is consistent across sociodemographic groups	7
	8b	D;E	If outcome assessment requires subjective interpretation, describe the qualifications and demographic characteristics of the outcome assessors	N/A
	8c	D;E	Report any actions to blind assessment of the outcome to be predicted	N/A
Predictors	9a	D	Describe the choice of initial predictors (e.g., literature, previous models, all available predictors) and any pre-selection of predictors before model building	7-8
	9b	D;E	Clearly define all predictors, including how and when they were measured (and any actions to blind assessment of predictors for the outcome and other predictors)	7-8
	9c	D;E	If predictor measurement requires subjective interpretation, describe the qualifications and demographic characteristics of the predictor assessors	N/A
Sample size	10	D;E	Explain how the study size was arrived at (separately for development and evaluation), and justify that the study size was sufficient to answer the research question. Include details of any sample size calculation	7
Missing data	11	D;E	Describe how missing data were handled. Provide reasons for omitting any data	8-9
Analytical methods	12a	D	Describe how the data were used (e.g., for development and evaluation of model performance) in the analysis, including whether the data were partitioned, considering any sample size requirements	7-10
	12b	D	Depending on the type of model, describe how predictors were handled in the analyses (functional form, rescaling, transformation, or any standardisation).	7-10
	12c	D	Specify the type of model, rationale[2], all model-building steps, including any hyperparameter tuning, and method for internal validation	8-10
	12d	D;E	Describe if and how any heterogeneity in estimates of model parameter values and model performance was handled and quantified across clusters (e.g., hospitals, countries). See TRIPOD-Cluster for additional considerations[3]	8-10
	12e	D;E	Specify all measures and plots used (and their rationale) to evaluate model performance (e.g., discrimination, calibration, clinical utility) and, if relevant, to compare multiple models	8-10
	12f	E	Describe any model updating (e.g., recalibration) arising from the model evaluation, either overall or for particular sociodemographic groups or settings	N/A
	12g	E	For model evaluation, describe how the model predictions were calculated (e.g., formula, code, object, application programming interface)	8-10
Class imbalance	13	D;E	If class imbalance methods were used, state why and how this was done, and any subsequent methods to recalibrate the model or the model predictions	N/A
Fairness	14	D;E	Describe any approaches that were used to address model fairness and their rationale	N/A
Model output	15	D	Specify the output of the prediction model (e.g., probabilities, classification). Provide details and rationale for any classification and how the thresholds were identified	8-10

[1] D=items relevant only to the development of a prediction model; E=items relating solely to the evaluation of a prediction model; D;E=items applicable to both the development and evaluation of a prediction model

[2] Separately for all model building approaches.

[3] TRIPOD-Cluster is a checklist of reporting recommendations for studies developing or validating models that explicitly account for clustering or explore heterogeneity in model performance (eg, at different hospitals or centres). Debray et al, BMJ 2023; 380: e071018 [DOI: 10.1136/bmj-2022-071018]

Training versus evaluation	16	D;E	Identify any differences between the development and evaluation data in healthcare setting, eligibility criteria, outcome, and predictors	8-10
Ethical approval	17	D;E	Name the institutional research board or ethics committee that approved the study and describe the participant-informed consent or the ethics committee waiver of informed consent	7
**OPEN SCIENCE**				
Funding	18a	D;E	Give the source of funding and the role of the funders for the present study	2
Conflicts of interest	18b	D;E	Declare any conflicts of interest and financial disclosures for all authors	1
Protocol	18c	D;E	Indicate where the study protocol can be accessed or state that a protocol was not prepared	18
Registration	18d	D;E	Provide registration information for the study, including register name and registration number, or state that the study was not registered	18
Data sharing	18e	D;E	Provide details of the availability of the study data	18
Code sharing	18f	D;E	Provide details of the availability of the analytical code[4]	18
**PATIENT & PUBLIC INVOLVEMENT**				
Patient & Public Involvement	19	D;E	Provide details of any patient and public involvement during the design, conduct, reporting, interpretation, or dissemination of the study or state no involvement.	N/A
**RESULTS**				
Participants	20a	D;E	Describe the flow of participants through the study, including the number of participants with and without the outcome and, if applicable, a summary of the follow-up time. A diagram may be helpful.	11
	20b	D;E	Report the characteristics overall and, where applicable, for each data source or setting, including the key dates, key predictors (including demographics), treatments received, sample size, number of outcome events, follow-up time, and amount of missing data. A table may be helpful. Report any differences across key demographic groups.	11
	20c	E	For model evaluation, show a comparison with the development data of the distribution of important predictors (demographics, predictors, and outcome).	11-13
Model development	21	D;E	Specify the number of participants and outcome events in each analysis (e.g., for model development, hyperparameter tuning, model evaluation)	11-13
Model specification	22	D	Provide details of the full prediction model (e.g., formula, code, object, application programming interface) to allow predictions in new individuals and to enable third-party evaluation and implementation, including any restrictions to access or re-use (e.g., freely available, proprietary)[5]	11-13
Model performance	23a	D;E	Report model performance estimates with confidence intervals, including for any key subgroups (e.g., sociodemographic). Consider plots to aid presentation.	11-13
	23b	D;E	If examined, report results of any heterogeneity in model performance across clusters. See TRIPOD Cluster for additional details[3].	N/A
Model updating	24	E	Report the results from any model updating, including the updated model and subsequent performance	N/A
**DISCUSSION**				
Interpretation	25	D;E	Give an overall interpretation of the main results, including issues of fairness in the context of the objectives and previous studies	14-16
Limitations	26	D;E	Discuss any limitations of the study (such as a non-representative sample, sample size, overfitting, missing data) and their effects on any biases, statistical uncertainty, and generalizability	15-16
Usability of the model in the context of current care	27a	D	Describe how poor quality or unavailable input data (e.g., predictor values) should be assessed and handled when implementing the prediction model	15-16
	27b	D	Specify whether users will be required to interact in the handling of the input data or use of the model, and what level of expertise is required of users	14-15
	27c	D;E	Discuss any next steps for future research, with a specific view to applicability and generalizability of the model	15

From: Collins GS, Moons KGM, Dhiman P, et al. *BMJ* 2024;385:e078378. doi:10.1136/bmj-2023-078378

[4] This relates to the analysis code, for example, any data cleaning, feature engineering, model building, evaluation.

[5] This relates to the code to implement the model to get estimates of risk for a new individual

Supplemental Table 1. Imputed values for features with missingness

Feature	Feature Type	Imputed
pH at 24 hours	Categorical	Normal
Systolic blood pressure	Categorical	Normal if no Vasopressor used at 24 hours, Abnormal if Vasopressor used at 24 hours
PaO2/FiO2	Categorical	No ARDS
Albumin	Continuous	4
Lactate	Continuous	0
Fluid balance	Continuous	0
Heart rate	Continuous	80
Temperature	Continuous	98.6
Bicarbonate	Continuous	24
Creatinine	Continuous	1
Blood glucose	Continuous	160
WBC	Continuous	8
Potassium	Continuous	4
Sodium	Continuous	140
HGB	Continuous	16 if Male, 14 if Female
HCT	Continuous	46 if Male, 42 if Female
PLT	Continuous	300

HCT: hematocrit; HGB: hemoglobin; PaO_2/FiO_2: ratio of arterial partial pressure of oxygen to fraction of inspired oxygen; PLT: platelet count; WBC: white blood cell count

Supplemental Table 2. Mortality prediction variables with definitions

Feature	Feature Type	Number (%) of missingness	Definition
Baseline Patient Characteristics			
Age	Continuous	0 (0)	N/A
Sex	Categorical	0 (0)	N/A
ICU Admission Information			
Admission diagnosis	Categorical	0 (0)	Burn, cardiovascular, dermatology, electrolyte abnormalities, endocrine, fever, gastrointestinal, hematologic, hepatic, infection, mental health, neoplasm, neurology, pneumonia, pregnancy, pulmonary, renal, respiratory, respiratory failure, sepsis, shock, syncope, toxicology/ingestion, trauma, weakness, or other
ICU Type	Categorical	0 (0)	Burn, cardiac, cardiothoracic, medical, neurosciences, surgical, or mixed
24 Hours After ICU Admission			
Severity of Illness			
APACHE II at 24 hours	Continuous	0 (0)	N/A
SOFA at 24 hours	Continuous	0 (0)	N/A
Vital Signs			
Heart rate at 24 hours	Continuous	3 (0.3)	Highest heart rate at 24 hours
Ind_miss heart rate at 24 hours			Indicator of missingness for heart rate at 24 hours
SBP at 24 hours	Categorical	12 (1.2)	Lowest systolic blood pressure at 24 hours. This variable is categorized into two levels by cut point 90
Ind_miss SBP at 24 hours			Indicator of missingness for SBP at 24 hours. This variable indicates missingness into two categories depending on the level of vasopressor at 24 hours
Temperature at 24 hours	Continuous	26 (2.6)	The most extreme temperature at 24 hours comparing with the normal value, 97.97
Ind_miss temperature at 24 hours			Indicator of missingness for temperature at 24 hours
ARDS Classification			
PaO_2:FiO_2 at 24 hours	Categorical	622 (62.8)	The lowest PaO_2:FiO_2 at 24 hours. This variable is categorized into four levels by cut points 100, 200, and 300.
Ind_miss PaO_2:FiO_2 at 24 hours			Indicator of missingness for PaO_2:FiO_2 at 24 hours
Supportive Care Devices			
CRRT at 24 hours	Categorical	0 (0)	
Mechanical ventilation at 24 hours	Categorical	0 (0)	N/A

Supplemental Table 2 (cont). Mortality prediction variables with definitions

Serum Laboratory Values			
Albumin at 24 hours	Continuous	521 (52.6)	The lowest albumin at 24 hours
Ind_miss albumin at 24 hours			Indicator of missingness for albumin at 24 hours
Bicarbonate at 24 hours	Continuous	46 (4.6)	The most extreme bicarbonate at 24 hours comparing with the normal value, 24
Ind_miss bicarbonate & creatinine & sodium at 24 hours			Indicator of missingness for bicarbonate & creatinine & sodium at 24 hours
Blood glucose at 24 hours	Continuous	31 (3.1)	The most extreme blood glucose at 24 hours comparing with the normal value, 125
Ind_miss blood glucose at 24 hours			Indicator of missingness for blood glucose at 24 hours
Creatinine at 24 hours	Continuous	46 (4.6)	The highest creatinine at 24 hours
HCT at 24 hours	Continuous	80 (8.1)	The lowest HCT at 24 hours
HGB at 24 hours	Continuous	80 (8.1)	The lowest HGB at 24 hours
Ind_miss HGB & HCT at 24 hours			Indicator of missingness for HGB & HCT at 24 hours. This variable indicates missingness into two categories depending on the level of Sex
Lactate at 24 hours	Continuous	670 (67.6)	The highest lactate at 24 hours
Ind_miss lactate at 24 hours			Indicator of missingness for lactate at 24 hours
pH at 24 hours	Categorical	582 (58.7)	The most extreme pH at 24 hours comparing with the normal value, 7.4. This variable is categorized into three levels by two cut points, 7.2 and 7.5
Ind_miss pH at 24 hours			Indicator of missingness for pH at 24 hours
PLT at 24 hours	Continuous	83 (8.4)	The most extreme PLT at 24 hours comparing with the normal value, 300.
Ind_miss PLT at 24 hours			Indicator of missingness for PLT at 24 hours
Potassium at 24 hours	Continuous	44 (4.4)	The most extreme potassium at 24 hours comparing with the normal value, 4.35
Ind_miss potassium at 24 hours			Indicator of missingness for potassium at 24 hours
Sodium at 24 hours	Continuous	46 (4.6)	The most extreme sodium at 24 hours comparing with the normal value, 140
WBC at 24 hours	Continuous	81 (8.2)	The most extreme WBC at 24 hours comparing with the normal value, 7.75
Ind_miss WBC at 24 hours			Indicator of missingness for WBC at 24 hours
Fluid balance at 24 hours	Continuous	100 (10.1)	
Ind_miss fluid balance at 24 hours			Indicator of missingness for fluid balance at 24 hours

Supplemental Table 2 (cont). Mortality prediction variables with definitions

Medication Data at 24 Hours			
MRC-ICU at 24 hours	Continuous	0 (0)	N/A
Vasopressor at 24 hours	Categorical	0 (0)	N/A
APACHE: Acute Physiology and Chronic Health Evaluation; CRRT: continuous renal replacement therapy; HCT: hematocrit; HGB: hemoglobin; MRC-ICU: Medication Regimen Complexity – Intensive Care Unit; PaO_2:FiO_2: ratio of partial pressure of arterial oxygen to fraction of inspired oxygen; PLT: platelets; SBP: systolic blood pressure; SOFA: Sequential Organ Failure Assessment; WBC: white blood cell count			

Supplemental Table 3. Full comparison of model variables in the training/test cohort for patients who died in the hospital vs those who did not

Variable	All (n=991)	Mortality (n=97)	No Mortality (n=894)	p-value
ICU baseline				
Age, mean (SD)	61.2 (17.6)	66.8 (15.5)	60.6 (17.7)	<0.01
Male sex, n (%)	563 (56.8)	56 (57.7)	507 (56.7)	0.93
ICU Type, n (%)				0.01
Medical ICU	404 (40.8)	53 (54.6)	351 (36.3)	
Cardiac/Cardiothoracic ICU	306 (30.9)	20 (20.6)	286 (32.0)	
Surgical ICU	97 (9.8)	4 (4.1)	93 (10.4)	
Other	184 (18.6)	20 (20.6)	164 (18.3)	
Primary ICU admission diagnosis, n (%)				<0.01
Cardiovascular	253 (25.5)	11 (11.3)	242 (27.0)	
Acute respiratory	124 (12.5)	25 (25.8)	99 (11.1)	
Neurological	121 (12.2)	12 (12.4)	109 (12.2)	
Sepsis	107 (10.8)	17 (17.5)	90 (10.1)	
Acute GI / Hepatic	83 (8.4)	8 (8.2)	75 (8.4)	
Other	303 (30.6)	24 (24.7)	279 (31.2)	
24 h after ICU admission				
Severity of illness, mean (SD)				
APACHE II Score	14.1 (6.4)	20.6 (6.0)	13.4 (6.0)	<0.01
SOFA Score	5.2 (4.2)	9.8 (3.9)	4.7 (3.9)	<0.01
Vital Signs				
Heart rate, mean (SD)	105.5 (21.6)	114.5 (25.0)	104.5 (21.0)	<0.01
Hypotension (SBP <90 mmHg), n (%)	316 (32.3)	47 (50.0)	269 (30.4)	<0.01
Temperature (F), mean (SD)	98.5 (3.9)	98.3 (2.5)	98.5 (4.0)	0.55
ARDS Classification, n (%)				<0.01
Mild	95 (25.8)	9 (12.5)	86 (29.0)	
Moderate	145 (39.3)	37 (51.4)	108 (36.4)	
Severe	46 (12.5)	14 (19.4)	32 (10.8)	
Supportive devices, n (%)				
CRRT at 24 h	11 (1.1)	3 (3.1)	8 (0.9)	0.15
MV at 24 h	291 (29.4)	55 (56.7)	236 (26.4)	<0.01
Serum laboratory values, mean (SD)				
Albumin mg/dL	2.9 (0.7)	2.5 (0.7)	3.0 (0.7)	<0.01
Bicarbonate mEq/L	23.7 (5.5)	22.4 (7.2)	23.8 (5.3)	0.06
Creatinine mg/dL	1.6 (2.0)	2.3 (2.1)	1.5 (2.0)	<0.01
Glucose mg/dL	158.5 (89.0)	173.8 (91.6)	156.8 (88.6)	0.09
Lactate mmol/L	2.6 (2.5)	3.8 (3.6)	2.3 (2.0)	<0.01
Potassium mEq/L	4.0 (2.8)	4.1 (0.9)	4.0 (0.7)	0.24
pH < 7.2, n (%)	46 (11.3)	18 (23.7)	28 (8.4)	<0.01
pH > 7.5, n (%)	32 (7.8)	11 (14.5)	21 (6.3)	<0.01
Sodium mEq/L	138.5 (5.7)	138.8 (7.9)	138.5 (5.4)	0.71
Hemoglobin g/dL	10.9 (2.4)	9.8 (2.4)	11.1 (2.4)	<0.01
Hematocrit %	33.2 (7.1)	30.3 (7.1)	33.6 (7.0)	<0.01
Platelets x 10^3/µL	207.4 (114.5)	197.8 (177.1)	208.5 (105.0)	0.57
White blood cells x 10^3/µL	11.8 (6.6)	14.8 (10.4)	11.5 (5.9)	<0.01
Fluid balance at 24 h (L), mean (SD)	0.7 (2.4)	0.9 (2.2)	0.7 (2.5)	0.43
Medications				
MRC-ICU score, mean (SD)	10.3 (7.7)	14.3 (8.3)	9.9 (7.5)	<0.01
Vasopressor use at 24 h, n (%)	231 (23.3)	46 (47.4)	185 (20.7)	<0.01

Supplemental Table 3 (cont). Comparison of model variables in the training/test cohort for patients who died in the hospital vs those who did not – full demographic information

**Note: Table excludes patients with missing data*
APACHE: Acute Physiology and Chronic Health Evaluation; ARDS: Acute Respiratory Distress Syndrome; CRRT: continuous renal replacement therapy; GI: gastrointestinal; ICU: intensive care unit; MRC-ICU: Medication Regimen Complexity-ICU; MV: mechanical ventilation; SOFA: Sequential Organ Failure Assessment

Supplemental Table 4. AUROC for mortality prediction models on test set

	**AUROC**
APACHE II	0.72, 0.58-0.86
SOFA	0.81, 0.69-0.93
MRC-ICU + SOFA + APACHE II	0.83, 0.71-0.95
MRC-ICU + SOFA + APACHE II (with interactions)	0.83, 0.74-0.90
Linear Logistic	0.85, 0.74-0.96
Linear Logistic (Full)	0.86, 0.79-0.93
Nature Cubic Splines Logistic	0.86, 0.75-0.97
Smoothing Splines Logistic	0.85, 0.74-0.96
Local Linear Logistic	0.84, 0.72-0.96
Random Forest	0.83, 0.71-0.95
SVM	0.85, 0.74-0.96
XGBoost	0.82, 0.70-0.94
APACHE: acute physiology and chronic health evaluation; AUROC: area under the receiver operating characteristic; SOFA: sequential organ failure assessment	

Supplemental Table 5. Mortality prediction variables univariate and multivariable analysis

	Univariate			Multivariate		
**Variable**	**Odds Ratio**	**95% CI**	**p-value**	**Odds Ratio**	**95% CI**	**p-value**
ICU Baseline						
Age	1.02	1.01, 1.04	<0.01	1.02	0.99, 1.05	0.22
Sex	1.15	0.71, 1.84	0.57	2.33	1.02, 5.36	0.05
ICU Type						
Burn ICU	0.18	0.04, 0.89	0.04	3.09	0.08, 116.48	0.54
Cardiac ICU	0.25	0.07, 0.84	0.03	0.65	0.07, 5.93	0.71
Cardiothoracic ICU	0.00	0.00, Inf	0.98	89168.57	0.00, Inf	1.00
Medical ICU	0.49	0.15, 1.58	0.23	1.16	0.14, 9.52	0.89
Neurosciences ICU	0.46	0.12, 1.74	0.25	2.92	0.21, 40.53	0.42
Surgical ICU	0.14	0.03, 0.71	0.02	0.04	0.00, 1.14	0.06
Primary ICU admission diagnosis						
Burn	0.43	0.06, 3.27	0.41	1.84	0.03, 113.30	0.77
Cardiovascular	0.44	0.09, 2.18	0.31	0.42	0.03, 5.41	0.51
Dermatology	0.57	0.05, 6.90	0.66	0.50	0.01, 22.50	0.72
Electrolyte Abnormalities	0.57	0.05, 6.90	0.66	0.52	0.02, 13.60	0.69
Endocrine	0.00	0.00, Inf	0.99	0.00	0.00, Inf	0.99
Fever	2.83	0.19, 41.99	0.45	21.13	0.25, 1786.36	0.18
Gastrointestinal	0.59	0.10, 3.48	0.56	0.20	0.01, 3.26	0.26
Hematologic	6.80	0.95, 48.69	0.06	2.91	0.14, 59.18	0.49
Hepatic	4.25	0.45, 40.01	0.21	2.95	0.08, 105.83	0.55
Infection	0.43	0.04, 5.11	0.50	0.52	0.02, 13.46	0.70
Mental Health	0.00	0.00, Inf	1.00	0.00	0.0000, Inf	1.00
Neoplasm	1.10	0.18, 6.62	0.92	1.30	0.10, 16.64	0.84
Neurology	1.00	0.20, 4.98	1.00	0.78	0.06, 9.32	0.84
Pneumonia	1.89	0.23, 15.74	0.56	0.93	0.05, 17.58	0.96
Pregnancy	0.00	0.00, Inf	0.99	0.00	0.00, Inf	1.00
Pulmonary	2.13	0.44, 10.22	0.35	1.71	0.19, 15.65	0.64
Renal	1.00	0.13, 7.94	1.00	0.81	0.03, 19.26	0.90
Respiratory	1.06	0.08, 13.52	0.96	1.42	0.03, 58.90	0.85
Respiratory failure	4.25	0.73, 24.77	0.11	1.02	0.06, 17.13	0.99
Sepsis	1.47	0.26, 8.40	0.67	0.47	0.04, 5.38	0.54
Shock	2.13	0.25, 17.93	0.49	0.40	0.02, 9.70	0.57
Syncope	0.00	0.00, Inf	0.99	0.00	0.00, Inf	1.00
Toxicology/Ingestion	0.00	0.00, Inf	0.99	0.00	0.00, Inf	0.99
Trauma	0.75	0.11, 4.92	0.76	0.39	0.02, 9.24	0.56
Weakness	2.13	0.15, 29.66	0.58	0.31	0.00, 47.55	0.65

Supplemental Table 5 (cont). Mortality prediction variables univariate and multivariate analysis

24 h after ICU admission						
Severity of illness, mean (SD)						
APACHE II Score	1.18	1.14, 1.23	<0.01	1.10	0.98, 1.22	0.10
SOFA Score	1.30	1.23, 1.39	<0.01	1.36	1.10, 1.68	<0.01
Vital Signs						
Heart rate	1.02	1.01, 1.03	<0.01	1.02	1.00, 1.03	0.05
Systolic blood pressure abnormal	2.61	1.62, 4.21	<0.01	0.90	0.40, 2.05	0.80
Temperature	0.99	0.94, 1.03	0.50	1.03	0.91, 1.17	0.61
ARDS Classification						
Mild	0.63	0.23, 1.75	0.38	0.55	0.13, 2.37	0.42
Moderate	2.24	0.99, 5.08	0.05	2.63	0.72, 9.60	0.14
Severe	2.67	1.00, 7.13	0.05	1.15	0.24, 5.68	0.86
Supportive devices						
CRRT at 24 h	3.48	0.91, 13.41	0.07	0.34	0.02, 4.68	0.42
MV at 24 h	4.43	2.74, 7.17	<0.01	0.77	0.18, 3.40	0.73
Serum laboratory values						
Albumin mg/dL	0.29	0.18, 0.46	<0.01	0.56	0.25, 1.22	0.15
Bicarbonate mEq/L	0.93	0.89, 0.98	<0.01	0.96	0.90, 1.04	0.32
Creatinine mg/dL	1.11	1.01, 1.23	0.04	0.89	0.69, 1.15	0.36
Glucose mg/dL	1.00	1.00, 1.00	0.11	1.00	1.00, 1.00	0.81
Lactate mmol/L	1.19	1.06, 1.33	<0.01	0.89	0.71, 1.13	0.35
Potassium mEq/L	1.27	0.94, 1.71	0.12	0.74	0.48, 1.16	0.19
pH < 7.2	4.10	1.98, 8.50	<0.01	3.02	0.77, 11.82	0.11
pH > 7.5	2.38	0.97, 5.80	0.06	2.14	0.54, 8.52	0.28
Sodium mEq/L	0.98	0.94, 1.03	0.48	0.98	0.92, 1.04	0.45
Hemoglobin g/dL	0.79	0.71, 0.88	<0.01	0.24	0.10, 0.60	<0.01
Hematocrit %	0.93	0.90, 0.97	<0.01	1.51	1.11, 2.03	<0.01
Platelets x 10^3/μL	1.00	1.00, 1.00	0.68	1.00	1.00, 1.00	0.50
White blood cells x 10^3/μL	1.07	1.04, 1.11	<0.01	1.05	0.99, 1.11	0.10
Fluid balance at 24 h (mL)	1.04	0.97, 1.12	0.30	0.96	0.78, 1.18	0.70
Medications						
MRC-ICU	1.08	1.05, 1.10	<0.01	0.95	0.88, 1.03	0.22
Vasopressor at 24 h	4.02	2.50, 6.49	<0.01	0.57	0.19, 1.71	0.32

Data are presented as n (%) or mean ± standard deviation (SD) unless otherwise stated. APACHE: acute physiology and chronic health evaluation; CRRT: continuous renal replacement therapy; ICU: intensive care unit; MRC-ICU: medication regimen complexity-ICU; MV: mechanical ventilation; SOFA: sequential organ failure assessment

Supplemental Table 6. Regression models for mortality with linear predictors

**Linear predictors**				
	**OR**		**95% CI**	**p-value**
SOFA at 24 hours	1.28		1.19, 1.37	<0.01
Age	1.04		1.02, 1.06	<0.01
Indicator for missingness of temperature at 24 hours	5.21		1.62, 17.00	<0.01
Lowest albumin at 24 hours	0.41		0.28, 0.59	<0.01
Lowest HGB at 24 hours	0.41		0.24, 0.69	<0.01
Lowest HCT at 24 hours	1.36		1.14, 1.62	<0.01
**Nature cubic splines**				
	**OR**		**95% CI**	**p-value**
SOFA at 24 hours (Linear predictor)	1.24		1.11, 1.38	<0.01
APACHE II at 24 hours (Nature cubic spline with knots at 8.5 and 16.5)				
Basis 1	7.23*10^{2}		11.26, 3.86*10^{5}	0.04
Basis 2	5.61*10^{5}		0.51, 8.77*10^{17}	0.21
Basis 3	3.58*10^{2}		6.26, 5.10*10^{5}	0.03
Mechanical Ventilation at 24 hours	0.25		0.10, 0.63	<0.01
Indicator for missingness of temperature at 24 hours	5.94		1.81, 20.04	<0.01
Indicator for missingness of albumin at 24 hours	0.32		0.17, 0.60	<0.01
Lowest HGB at 24 hours (Nature cubic spline with knots at 12.5 and 14.1)				
Basis 1	0.00		0.00, 1.44*10^{-3}	<0.01
Basis 2	0.00		0.00, 9.33*10^{-4}	<0.01
Basis 3	0.43		0.00, 1.43*10^{4}	0.86
Lowest HCT at 24 hours (Nature cubic spline with knots at 40.6 and 42.4)				
Basis 1	2.50*10^{4}		84.54, 7.53*10^{6}	<0.01
Basis 2	1.86*10^{5}		0.00, 1.48*10^{13}	0.23
Basis 3	0.00		0.00, 6.67*10^{2}	0.24
**Smoothing splines**				
**ANOVA for parametric effects**	**Df**	**MSE**	**F-value**	**p-value**
SOFA at 24 hours	1	52.65	73.74	<0.01
Age	1	4.86	6.80	<0.01
Fluid balance at 24 hours (Smoothing spline with df=2)	1	0.67	0.93	0.33
Indicator for missingness of temperature at 24 hours	1	8.40	11.77	<0.01
Lowest albumin at 24 hours	1	29.39	41.17	<0.01
Lowest HGB at 24 hours	1	0.01	0.01	0.91
Lowest HCT at 24 hours	1	11.35	15.89	<0.01
Residuals	784	0.714		
**ANOVA for nonparametric effects**	**Df**		**Chisq-Value**	**p-value**
Fluid balance at 24 hours (Smoothing spline with df=2)	1		7.18	<0.01
**Local linear**				
**ANOVA for parametric effects**	**Df**	**MSE**	**F-value**	**p-value**
SOFA at 24 hours	1	53.99	74.54	<0.01
Age	1	4.93	6.81	<0.01
Fluid balance at 24 hours (local linear predictor)	1	0.58	0.81	0.37
Indicator for missingness of temperature at 24 hours	1	9.01	12.44	<0.01
Lowest albumin at 24 hours	1	29.94	41.34	<0.01
Lowest HGB at 24 hours	1	0.02	0.02	0.88
Lowest HCT at 24 hours	1	11.43	15.77	<0.01
Residuals	783.69	0.724		
**ANOVA for nonparametric effects**	**Df**		**Chisq-Value**	**p-value**
Fluid balance at 24 hours (Smoothing spline with df=2)	1.3		10.31	<0.01
APACHE: acute physiology and chronic health evaluation; HCT: hematocrit; HGB: hemoglobin; SOFA: sequential organ failure assessment				

Supplemental Table 7. UNC 5000 Validation cohort demographics

	All (n=4878)	Mortality (n=964)	No mortality (n=3914)	p-value
ICU baseline				
Age, mean (SD)	58.9 (16.5)	62.5 (14.5)	58.0 (16.8)	0.00
Male sex, n (%)	2532 (51.9)	528 (54.8)	2004 (51.2)	0.05
ICU Type, n (%)				0.57
Medical ICU	4631 (94.9)	917 (95.1)	3714 (94.9)	
Surgical ICU	126 (2.6)	23 (2.4)	103 (2.6)	
Cardiac/Cardiothoracic ICU	56 (1.1)	15 (1.6)	41 (1.0)	
Other	65 (1.3)	9 (0.9)	56 (1.4)	
Primary ICU admission diagnosis, n (%)				0.00
Acute Respiratory	1052 (21.6)	247 (25.6)	805 (20.6)	
Sepsis	958 (19.6)	250 (25.9)	708 (18.1)	
Acute GI / Hepatic	761 (15.6)	142 (14.7)	619 (15.8)	
Hematology / Oncology	482 (9.9)	104 (10.8)	378 (9.7)	
Cardiovascular	364 (7.5)	58 (6.0)	306 (7.8)	
Neurology	331 (6.8)	57 (5.9)	274 (7.0)	
Other	930 (19.1)	106 (11.0)	824 (21.1)	
24 h after ICU admission				
Severity of illness, mean (SD)				
APACHE II Score	15.9 (6.3)	18.8 (6.4)	15.2 (6.0)	0.00
SOFA Score	7.0 (4.5)	10.3 (4.7)	6.2 (4.0)	0.00
Vital Signs				
Heart rate, mean (SD)	110.1 (21.8)	115.9 (24.2)	108.7 (20.9)	0.00
Hypotension (SBP < 90 mmHg), n (%)	1305 (31.4)	285 (41.7)	1020 (29.3)	0.00
Temperature (F), mean (SD)	98.7 (2.7)	98.5 (4.7)	98.8 (1.9)	0.17
ARDS Classification, n (%)				0.00
Mild	386 (22.1)	104 (15.8)	282 (26.0)	
Moderate	667 (38.3)	265 (40.2)	402 (37.1)	
Severe	408 (23.4)	227 (34.4)	181 (16.7)	
Supportive devices, n (%)				
CRRT at 24 h	611 (12.5)	316 (32.8)	295 (7.5)	0.00
MV at 24 h	1415 (29.0)	504 (52.3)	911 (23.3)	0.00
Serum laboratory values, mean (SD)				
Albumin mg/dL	2.6 (0.5)	2.5 (0.5)	2.7 (0.5)	0.00
Bicarbonate mEq/L	22.7 (6.5)	20.6 (7.5)	23.2 (6.2)	0.00
Creatinine mg/dL	2.0 (2.4)	2.5 (2.1)	1.9 (2.4)	0.00
Glucose mg/dL	154.4 (93.3)	165.7 (96.3)	151.6 (92.3)	0.00
Lactate mmol/L	3.3 (4.3)	5.2 (5.8)	2.2 (2.3)	0.00
Potassium mEq/L	4.1 (0.8)	4.4 (1.0)	4.1 (0.8)	0.00
pH < 7.2, n (%)	191 (9.5)	144 (20.1)	47 (3.6)	0.00
pH > 7.5, n (%)	142 (7.1)	33 (4.6)	109 (8.4)	0.00
Sodium mEq/L	137.1 (6.5)	137.4 (6.8)	137.1 (6.4)	0.25
Hemoglobin g/dL	9.7 (2.3)	9.4 (2.3)	9.8 (2.2)	0.00
Hematocrit %	30.21 (7.0)	29.4 (7.3)	30.4 (6.9)	0.00
Platelets x 10^3/µL	199.6 (136.9)	180.8 (146.1)	204.3 (134.1)	0.00
White blood cells x 10^3/µL	13.4 (18.6)	17.2 (19.9)	12.5 (18.2)	0.00
Fluid balance at 24 h (L), mean (SD)	1.8 (3.0)	2.5 (3.3)	1.6 (2.9)	0.00

Supplemental Table 7 (cont). UNC 5000 Validation cohort demographics

Medications				
MRC-ICU, mean (SD)	9.9 (6.6)	13.8 (7.7)	9.0 (6.0)	0.00
Vasopressor at 24 h, n (%)	2106 (43.2)	659 (68.4)	1447 (37.0)	0.00
**Note: Table excludes patients with missing data*<br>APACHE: Acute Physiology and Chronic Health Evaluation; ARDS: Acute Respiratory Distress Syndrome; CRRT: continuous renal replacement therapy; ICU: intensive care unit; MRC-ICU: Medication Regimen Complexity-ICU; MV: mechanical ventilation; SOFA: Sequential Organ Failure Assessment				

Supplemental Table 8. Data missingness in UNC 5000 validation set

Feature	Number (%) Missingness
Age	0 (0)
Sex	0 (0)
MRC-ICU score at 24 hours	0 (0)
ICU type	0 (0)
Mechanical ventilation at 24 hours	0 (0)
Fluid balance at 24 hours	0 (0)
Admission diagnosis	0 (0)
Fluid balance at 24 hours	4 (0.1)
CRRT at 24 hours	0 (0)
Vasopressor at 24 hours	0 (0)
Heart rate at 24 hours	3 (0.1)
SBP at 24 hours	716 (14.7)
Temperature at 24 hours	499 (10.2)
pH at 24 hours	2865 (58.7)
Bicarbonate at 24 hours	222 (4.6)
Creatinine at 24 hours	192 (3.9)
Blood glucose at 24 hours	124 (2.5)
WBC at 24 hours	295 (6.1)
Lactate at 24 hours	3095 (63.5)
Potassium at 24 hours	192 (3.9)
Sodium at 24 hours	183 (3.8)
Albumin at 24 hours	2033 (41.7)
HGB at 24 hours	269 (5.5)
HCT at 24 hours	276 (5.7)
PLT at 24 hours	293 (6.0)
PaO_2:FiO_2 at 24 hours	3134 (64.3)

CRRT: continuous renal replacement therapy; HCT: hematocrit; HGB: hemoglobin; ICU: Intensive care unit; MRC-ICU: Medication Regimen Complexity-Intensive Care Unit; PaO_2:FiO_2: ratio of arterial partial pressure of oxygen to fraction of inspired oxygen; PLT: platelet count; SBP: systolic blood pressure; WBC: white blood cell count

Supplemental Table 9. AUROC for mortality prediction models on UNC 5000 validation set

	**AUROC**
APACHE II	0.66, 0.64-0.68
SOFA	0.75, 0.73-0.77
MRC-ICU + SOFA + APACHE II	0.72, 0.70-0.74
MRC-ICU + SOFA + APACHE II (with interactions)	0.71, 0.69-0.73
Linear Logistic	0.75, 0.73-0.77
Linear Logistic (Full)	0.71, 0.69-0.73
Nature Cubic Splines Logistic	0.69, 0.67-0.71
Smoothing Splines Logistic	0.73, 0.71-0.75
Local Linear Logistic	0.73, 0.71-0.75
Random Forest	0.78, 0.76-0.80
SVM	0.74, 0.72-0.76
XGBoost	0.73, 0.71-0.75
APACHE: acute physiology and chronic health evaluation; AUROC: area under the receiver operating characteristic; SOFA: sequential organ failure assessment	

Supplemental Table 10. Accuracy, sensitivity, specificity, negative predictive value, and positive predictive value for mortality prediction on UNC 5000 validation set

**Maximizing INF**	**Accuracy**	**Sensitivity**	**Specificity**	**PPV**	**NPV**
APACHE II	0.67, 0.65-0.68	0.49, 0.45-0.52	0.71, 0.70-0.73	0.29, 0.27-0.32	0.85, 0.84-0.86
SOFA	0.56, 0.55-0.58	0.83, 0.80-0.85	0.50, 0.48-0.52	0.29, 0.27-0.31	0.92, 0.91-0.93
MRC-ICU + SOFA + APACHE II	0.67, 0.65-0.68	0.65, 0.62-0.68	0.67, 0.66-0.69	0.33, 0.31-0.35	0.89, 0.87-0.90
MRC-ICU + SOFA + APACHE II (with interactions)	0.64, 0.63-0.66	0.68, 0.65-0.71	0.63, 0.62-0.65	0.31, 0.29-0.33	0.89, 0.88-0.90
Linear Logistic	0.66, 0.64-0.67	0.71, 0.68-0.74	0.64, 0.63-0.66	0.33, 0.31-0.35	0.90, 0.89-0.91
Linear Logistic (Full)	0.64, 0.62-0.65	0.69, 0.66-0.72	0.62, 0.61-0.64	0.31, 0.29-0.33	0.89, 0.88-0.9
Nature Cubic Splines Logistic	0.63, 0.61-0.64	0.65, 0.62-0.68	0.62, 0.61-0.64	0.30, 0.28-0.32	0.88, 0.87-0.89
Smoothing Splines Logistic	0.67, 0.66-0.68	0.67, 0.64-0.70	0.67, 0.65-0.68	0.33, 0.31-0.35	0.89, 0.88-0.90
Local Linear Logistic	0.68, 0.66-0.69	0.65, 0.62-0.68	0.68, 0.67-0.70	0.34, 0.31-0.36	0.89, 0.88-0.90
Random Forest	0.55, 0.53-0.56	0.88, 0.86-0.90	0.46, 0.45-0.48	0.29, 0.27-0.30	0.94, 0.93-0.95
SVM	0.66, 0.64-0.67	0.72, 0.69-0.75	0.64, 0.63-0.66	0.33, 0.31-0.35	0.90, 0.89-0.91
XGBoost	0.74, 0.73-0.75	0.50, 0.47-0.53	0.80, 0.79-0.81	0.38, 0.36-0.41	0.87, 0.86-0.88
**Maximizing MCC**	**Accuracy**	**Sensitivity**	**Specificity**	**PPV**	**NPV**
APACHE II	0.67, 0.65-0.68	0.49, 0.45-0.52	0.71, 0.70-0.73	0.29, 0.27-0.32	0.85, 0.84-0.86
SOFA	0.75, 0.73-0.76	0.56, 0.53-0.59	0.79, 0.78-0.81	0.40, 0.37-0.43	0.88, 0.87-0.89
MRC-ICU + SOFA + APACHE II	0.68, 0.66-0.69	0.64, 0.61-0.67	0.69, 0.67-0.70	0.33, 0.31-0.36	0.89, 0.87-0.90
MRC-ICU + SOFA + APACHE II (with interactions)	0.69, 0.67-0.70	0.61, 0.58-0.64	0.71, 0.69-0.72	0.34, 0.32-0.36	0.88, 0.87-0.89
Linear Logistic	0.71, 0.70-0.72	0.64, 0.61-0.67	0.72, 0.71-0.74	0.36, 0.34-0.39	0.89, 0.88-0.90
Linear Logistic (Full)	0.70, 0.69-0.71	0.59, 0.55-0.62	0.73, 0.71-0.74	0.35, 0.32-0.37	0.88, 0.87-0.89
Nature Cubic Splines Logistic	0.63, 0.61-0.64	0.65, 0.62-0.68	0.62, 0.61-0.64	0.30, 0.28-0.32	0.88, 0.87-0.89
Smoothing Splines Logistic	0.75, 0.74-0.76	0.49, 0.46-0.53	0.81, 0.80-0.83	0.39, 0.37-0.42	0.87, 0.86-0.88
Local Linear Logistic	0.75, 0.74-0.77	0.47, 0.44-0.51	0.82, 0.81-0.83	0.40, 0.37-0.43	0.86, 0.85-0.87
Random Forest	0.67, 0.66-0.69	0.75, 0.72-0.78	0.65, 0.64-0.67	0.35, 0.33-0.37	0.91, 0.90-0.92
SVM	0.77, 0.75-0.78	0.49, 0.45-0.52	0.83, 0.82-0.85	0.42, 0.39-0.45	0.87, 0.86-0.88
XGBoost	0.78, 0.76-0.79	0.36, 0.33-0.39	0.88, 0.87-0.89	0.42, 0.39-0.46	0.85, 0.84-0.86
**Maximizing F1**	**Accuracy**	**Sensitivity**	**Specificity**	**PPV**	**NPV**
APACHE II	0.67, 0.65-0.68	0.49, 0.45-0.52	0.71, 0.70-0.73	0.29, 0.27-0.32	0.85, 0.84-0.86
SOFA	0.75, 0.73-0.76	0.56, 0.53-0.59	0.79, 0.78-0.81	0.40, 0.37-0.43	0.88, 0.87-0.89
MRC-ICU + SOFA + APACHE II	0.69, 0.68-0.70	0.61, 0.58-0.64	0.71, 0.69-0.72	0.34, 0.32-0.36	0.88, 0.87-0.89

Supplemental Table 10 (cont). Accuracy, sensitivity, specificity, negative predictive value, and positive predictive value for mortality prediction on UNC 5000 validation set

MRC-ICU + SOFA + APACHE II (with interactions)	0.70, 0.69-0.71	0.57, 0.54-0.6	0.73, 0.72-0.75	0.35, 0.32-0.37	0.87, 0.86-0.89
Linear Logistic	0.71, 0.70-0.73	0.63, 0.60-0.66	0.74, 0.72-0.75	0.37, 0.35-0.39	0.89, 0.88-0.90
Linear Logistic (Full)	0.70, 0.69-0.71	0.59, 0.55-0.62	0.73, 0.71-0.74	0.35, 0.32-0.37	0.88, 0.87-0.89
Nature Cubic Splines Logistic	0.63, 0.62-0.65	0.64, 0.61-0.67	0.63, 0.62-0.65	0.30, 0.28-0.32	0.88, 0.86-0.89
Smoothing Splines Logistic	0.75, 0.74-0.76	0.49, 0.46-0.53	0.81, 0.80-0.83	0.39, 0.37-0.42	0.87, 0.86-0.88
Local Linear Logistic	0.75, 0.74-0.77	0.47, 0.44-0.51	0.82, 0.81-0.83	0.40, 0.37-0.43	0.86, 0.85-0.87
Random Forest	0.77, 0.76-0.78	0.59, 0.55-0.62	0.81, 0.80-0.82	0.44, 0.41-0.46	0.89, 0.88-0.90
SVM	0.77, 0.75-0.78	0.49, 0.45-0.52	0.83, 0.82-0.85	0.42, 0.39-0.45	0.87, 0.86-0.88
XGBoost	0.78, 0.76-0.79	0.36, 0.33-0.39	0.88, 0.87-0.89	0.42, 0.39-0.46	0.85, 0.84-0.86

INF: Informedness; MCC: Matthew's Correlation Coefficient; PPV: positive predictive value; NPV: negative predictive value; APACHE II: Acue Physiology and Chronic Health Evaluation II; SOFA: Sequential Organ Failure Assessment; MRC-ICU: Medication Regimen Complexity-Intensive Care Unit; SVM: Support Vector Machine

Supplemental Table 11. External validation cohort demographics (OHSU)

	All (n=12,290)	Mortality (n=1,259)	No mortality (n=11,031)	p-value
ICU baseline				
Age, mean (SD)	60.1 (17.5)	64.3 (15.6)	59.7 (17.6)	0.00
Male sex, n (%)	7295 (59.4)	773 (61.4)	6522 (59.1)	0.13
ICU Type, n (%)				0.00
Medical ICU	4033 (32.8)	363 (28.8)	3670 (33.3)	
Surgical ICU	3616 (29.4)	264 (21.0)	3352 (30.4)	
Neurosciences ICU	2681 (21.8)	225 (17.9)	2456 (22.3)	
Cardiac/Cardiothoracic ICU	1960 (16.0)	407 (32.3)	1553 (14.1)	
Primary ICU admission diagnosis, n (%)				0.00
Neurological	2757 (22.4)	252 (20.0)	2505 (22.7)	
Cardiovascular	2661 (21.7)	204 (16.2)	2457 (22.3)	
Trauma	825 (6.7)	44 (3.5)	781 (7.1)	
Acute Respiratory	486 (4.0)	88 (7.0)	398 (3.6)	
Sepsis	302 (2.5)	54 (4.3)	248 (2.23)	
Hematologic / Oncology	251 (2.0)	66 (5.2)	185 (1.7)	
Acute GI / Hepatic	212 (1.7)	38 (3.0)	174 (1.56)	
Other	4796 (39.0)	513 (40.8)	4283 (38.8)	
24 h after ICU admission				
Severity of illness, mean (SD)				
APACHE II Score	9.1 (4.1)	12.4 (4.8)	8.7 (3.9)	0.00
SOFA Score	4.6 (3.6)	7.4 (3.4)	4.3 (3.4)	0.00
Vital Signs				
Heart rate, mean (SD)	107.0 (21.7)	114.3 (23.9)	106.2 (21.3)	0.00
Hypotension (SBP < 90 mmHg), n (%)	11518 (95.0)	1173 (96.0)	10345 (94.8)	0.09
Temperature (F), mean (SD)	36.22 (1.55)	35.8 (2.1)	36.3 (1.5)	0.00
ARDS Classification, n (%)				0.00
Mild	1293 (24.6)	202 (20.5)	1091 (25.6)	
Moderate	1353 (25.8)	278 (28.3)	1075 (25.2)	
Severe	863 (16.4)	289 (29.4)	574 (13.5)	
Supportive devices, n (%)				
CRRT at 24 h	592 (4.8)	290 (23.0)	302 (2.7)	0.00
MV at 24 h	6903 (56.2)	1023 (81.3)	5880 (53.3)	0.00
Serum laboratory values, mean (SD)				
Albumin mg/dL	2.7 (0.7)	2.3 (0.7)	2.8 (0.7)	0.00
Bicarbonate mEq/L	23.9 (5.3)	22.3 (6.6)	24.0 (5.1)	0.00
Creatinine mg/dL	1.4 (1.6)	1.8 (1.5)	1.4 (1.6)	0.00
Glucose mg/dL	160.6 (87.2)	182.2 (101.1)	158.1 (85.1)	0.00
Lactate mmol/L	3.3 (3.0)	4.5 (4.3)	3.1 (2.6)	0.00
Potassium mEq/L	4.1 (0.9)	4.2 (1.0)	4.1 (0.8)	0.00
pH < 7.2, n (%)	964 (9.8)	257 (21.5)	707 (8.2)	0.00
pH > 7.5, n (%)	498 (5.1)	78 (6.5)	420 (4.9)	0.00
Sodium mEq/L	137.7 (5.9)	137.9 (7.7)	137.7 (5.7)	0.37
Hemoglobin g/dL	10.8 (2.4)	10.1 (2.6)	10.8 (2.4)	0.00
Hematocrit %	33.1 (7.6)	31.1 (8.1)	33.4 (7.4)	0.00
Platelets x $10^3/\mu L$	193.8 (108.6)	177.3 (127.1)	195.7 (106.1)	0.00
White blood cells x $10^3/\mu L$	14.0 (17.8)	17.1 (22.5)	13.6 (17.1)	0.00
Fluid balance at 24 h (L), mean (SD)	0.4 (1.6)	0.8 (2.1)	0.3 (1.6)	0.00

Supplemental Table 11 (cont). External validation cohort demographics (OHSU)

Medications				
MRC-ICU, mean (SD)	5.3 (4.0)	7.8 (5.0)	7.8 (5.0)	0.00
Vasopressor at 24 h, n (%)	4016 (32.7)	739 (58.8)	3277 (29.7)	0.00

**Note: Table excludes patients with missing data*
***Not applicable (N/A): Burn and mixed ICUs are not present at OHSU*
APACHE: acute physiology and chronic health evaluation; CRRT: continuous renal replacement therapy; ICU: intensive care unit; MRC-ICU: medication regimen complexity-ICU; MV: mechanical ventilation; SBP: systolic blood pressure; SOFA: sequential organ failure assessment

Supplemental Table 12. Data missingness in OHSU validation set

Feature	Number (%) Missingness
Age	7 (6e-04)
Sex	0 (0)
MRC-ICU score at 24 hours	609 (0.05)
ICU type	0 (0)
Mechanical ventilation at 24 hours	0 (0)
Fluid balance at 24 hours	68 (0.01)
Admission diagnosis	0 (0)
CRRT at 24 hours	0 (0)
Vasopressor at 24 hours	3 (2e-04)
Heart rate at 24 hours	90 (0.01)
SBP at 24 hours	159 (0.01)
Temperature at 24 hours	94 (0.01)
pH at 24 hours	2442 (0.20)
Bicarbonate at 24 hours	231 (0.02)
Creatinine at 24 hours	236 (0.02)
Blood glucose at 24 hours	123 (0.01)
WBC at 24 hours	397 (0.03)
Lactate at 24 hours	7035 (0.57)
Potassium at 24 hours	229 (0.02)
Sodium at 24 hours	223 (0.02)
Albumin at 24 hours	790 (0.06)
HGB at 24 hours	379 (0.03)
HCT at 24 hours	9700 (0.79)
PLT at 24 hours	396 (0.03)
PaO_2:FiO_2 at 24 hours	7038 (0.57)

CRRT: continuous renal replacement therapy; HCT: hematocrit; HGB: hemoglobin; ICU: Intensive care unit; MRC-ICU: Medication Regimen Complexity-Intensive Care Unit; PaO_2:FiO_2: ratio of arterial partial pressure of oxygen to fraction of inspired oxygen; PLT: platelet count; SBP: systolic blood pressure; WBC: white blood cell count

Supplemental Table 13. Accuracy, sensitivity, specificity, negative predictive value, and positive predictive value for mortality prediction models on OHSU set

Maximizing INF	**Accuracy**	**Sensitivity**	**Specificity**	**PPV**	**NPV**
APACHE II	0.90, 0.89-0.90	0.11, 0.09-0.13	0.98, 0.98-0.99	0.44, 0.38-0.49	0.91, 0.90-0.91
SOFA	0.67, 0.66-0.67	0.70, 0.67-0.72	0.66, 0.65-0.67	0.19, 0.18-0.20	0.95, 0.95-0.96
MRC-ICU + SOFA + APACHE II	0.85, 0.85-0.86	0.34, 0.31-0.37	0.91, 0.90-0.91	0.30, 0.27-0.32	0.92, 0.92-0.93
MRC-ICU + SOFA + APACHE II (with interactions)	0.84, 0.84-0.85	0.37, 0.35-0.4	0.89, 0.89-0.9	0.28, 0.26-0.31	0.93, 0.92-0.93
Linear Logistic	0.73, 0.72-0.74	0.65, 0.62-0.67	0.74, 0.73-0.75	0.22, 0.21-0.23	0.95, 0.94-0.95
Linear Logistic (Full)	0.21, 0.2-0.21	0.84, 0.82-0.86	0.14, 0.13-0.14	0.1, 0.09-0.1	0.88, 0.87-0.9
Nature Cubic Splines Logistic	0.86, 0.85-0.86	0.33, 0.30-0.35	0.92, 0.91-0.92	0.31, 0.28-0.33	0.92, 0.92-0.93
Smoothing Splines Logistic	0.78, 0.77-0.79	0.53, 0.50-0.55	0.81, 0.80-0.82	0.24, 0.22-0.25	0.94, 0.93-0.94
Local Linear Logistic	0.82, 0.81-0.83	0.41, 0.38-0.44	0.87, 0.86-0.87	0.26, 0.24-0.28	0.93, 0.92-0.93
Random Forest	0.38, 0.37-0.39	0.95, 0.94-0.96	0.32, 0.31-0.33	0.14, 0.13-0.14	0.98, 0.98-0.99
SVM	0.81, 0.8-0.81	0.48, 0.46-0.51	0.84, 0.83-0.85	0.25, 0.24-0.27	0.94, 0.93-0.94
XGBoost	0.85, 0.84-0.85	0.43, 0.4-0.46	0.89, 0.89-0.9	0.26, 0.24-0	0.93, 0.93-0.94
**Maximizing MCC**					
APACHE II	0.90, 0.89-0.90	0.11, 0.09-0.13	0.98, 0.98-0.99	0.44, 0.38-0.49	0.91, 0.90-0.91
SOFA	0.85, 0.84-0.86	0.29, 0.27-0.32	0.91, 0.91-0.92	0.27, 0.25-0.30	0.90, 0.91-0.92
MRC-ICU + SOFA + APACHE II	0.86, 0.85-0.86	0.32, 0.29-0.34	0.92, 0.91-0.92	0.30, 0.28-0.33	0.92, 0.92-0.93
MRC-ICU + SOFA + APACHE II (with interactions)	0.87, 0.86-0.87	0.28, 0.26-0.31	0.93, 0.93-0.94	0.32, 0.29-0.35	0.92, 0.92-0.93
Linear Logistic	0.85, 0.84-0.85	0.38, 0.35-0.41	0.90, 0.89-0.90	0.29, 0.27-0.32	0.93, 0.92-0.93
Linear Logistic (Full)	0.21, 0.21-0.22	0.82, 0.8-0.85	0.14, 0.14-0.15	0.1, 0.09-0.1	0.88, 0.86-0.89
Nature Cubic Splines Logistic	0.90, 0.89-0.90	0.06, 0.05-0.08	0.99, 0.99-0.99	0.45, 0.37-0.52	0.90, 0.90-0.91
Smoothing Splines Logistic	0.87, 0.87-0.88	0.25, 0.22-0.27	0.94, 0.94-0.95	0.32, 0.29-0.35	0.92, 0.91-0.92
Local Linear Logistic	0.88, 0.87-0.88	0.22, 0.20-0.25	0.95, 0.95-0.96	0.34, 0.31-0.37	0.92, 0.91-0.92
Random Forest	0.68, 0.67-0.69	0.77, 0.75-0.8	0.67, 0.66-0.68	0.21, 0.2-0.22	0.96, 0.96-0.97
SVM	0.81, 0.8-0.81	0.48, 0.46-0.51	0.84, 0.83-0.85	0.25, 0.24-0.27	0.94, 0.93-0.94
XGBoost	0.89, 0.88-0.89	0.26, 0.24-0.29	0.96, 0.95-0.96	0.4, 0.37-0.44	0.92, 0.92-0.93
**Maximizing F1**					

Supplemental Table 13 (cont). Accuracy, sensitivity, specificity, negative predictive value, and positive predictive value for mortality prediction models on OHSU set

APACHE II	0.90, 0.89-0.90	0.11, 0.09-0.13	0.98, 0.98-0.99	0.44, 0.38-0.49	0.91, 0.90-0.91
SOFA	0.85, 0.84-0.86	0.29, 0.27-0.32	0.91, 0.91-0.92	0.27, 0.25-0.30	0.92, 0.91-0.92
MRC-ICU + SOFA + APACHE II	0.86, 0.86-0.87	0.28, 0.26-0.31	0.93, 0.92-0.93	0.31, 0.28-0.34	0.92, 0.92-0.93
MRC-ICU + SOFA + APACHE II (with interactions)	0.87, 0.87-0.88	0.23, 0.21-0.26	0.95, 0.94-0.95	0.32, 0.29-0.35	0.92, 0.91-0.92
Linear Logistic	0.85, 0.84-0.85	0.38, 0.35-0.41	0.90, 0.89-0.90	0.29, 0.27-0.32	0.93, 0.92-0.93
Linear Logistic (Full)	0.21, 0.21-0.22	0.82, 0.8-0.85	0.14, 0.14-0.15	0.1, 0.09-0.1	0.88, 0.86-0.89
Nature Cubic Splines Logistic	0.90, 0.89-0.90	0.06, 0.05-0.08	0.99, 0.99-0.99	0.45, 0.37-0.52	0.90, 0.90-0.91
Smoothing Splines Logistic	0.87, 0.87-0.88	0.25, 0.22-0.27	0.94, 0.94-0.95	0.32, 0.29-0.35	0.92, 0.91-0.92
Local Linear Logistic	0.88, 0.87-0.88	0.22, 0.20-0.25	0.95, 0.95-0.96	0.34, 0.31-0.37	0.92, 0.91-0.92
Random Forest	0.72, 0.72-0.73	0.73, 0.7-0.75	0.72, 0.72-0.73	0.23, 0.21-0.24	0.96, 0.95-0.96
SVM	0.81, 0.8-0.81	0.48, 0.46-0.51	0.84, 0.83-0.85	0.25, 0.24-0.27	0.94, 0.93-0.94
XGBoost	0.89, 0.88-0.89	0.26, 0.24-0.29	0.96, 0.95-0.96	0.4, 0.37-0.44	0.92, 0.92-0.93
INF: Informedness; MCC: Matthew's Correlation Coefficient; PPV: positive predictive value; NPV: negative predictive value; APACHE II: Acue Physiology and Chronic Health Evaluation II; SOFA: Sequential Organ Failure Assessment; MRC-ICU: Medication Regimen Complexity-Intensive Care Unit; SVM: Support Vector Machine					

Supplemental Table 14. AUROC for mortality prediction models on OHSU validation set

	**AUROC**
APACHE II	0.73, 0.71-0.75
SOFA	0.73, 0.71-0.75
MRC-ICU + SOFA + APACHE II	0.75, 0.73-0.77
MRCICU + SOFA + APACHE II (With Interactions)	0.76, 0.74-0.78
Linear Logistic	0.77, 0.75-0.79
Linear Logistic (Full)	0.54, 0.52-0.56
Nature Cubic Splines Logistic	0.73, 0.71-0.75
Smoothing Splines Logistic	0.77, 0.75-0.79
Local Linear Logistic	0.76, 0.74-0.78
Random Forest	0.80, 0.79-0.81
SVM	0.75, 0.74-0.76
XGBoost	0.78, 0.77-0.79
AUROC: area under the receiver operating characteristic	

Supplemental Figure 1. Feature importance graph for Random Forest on test set

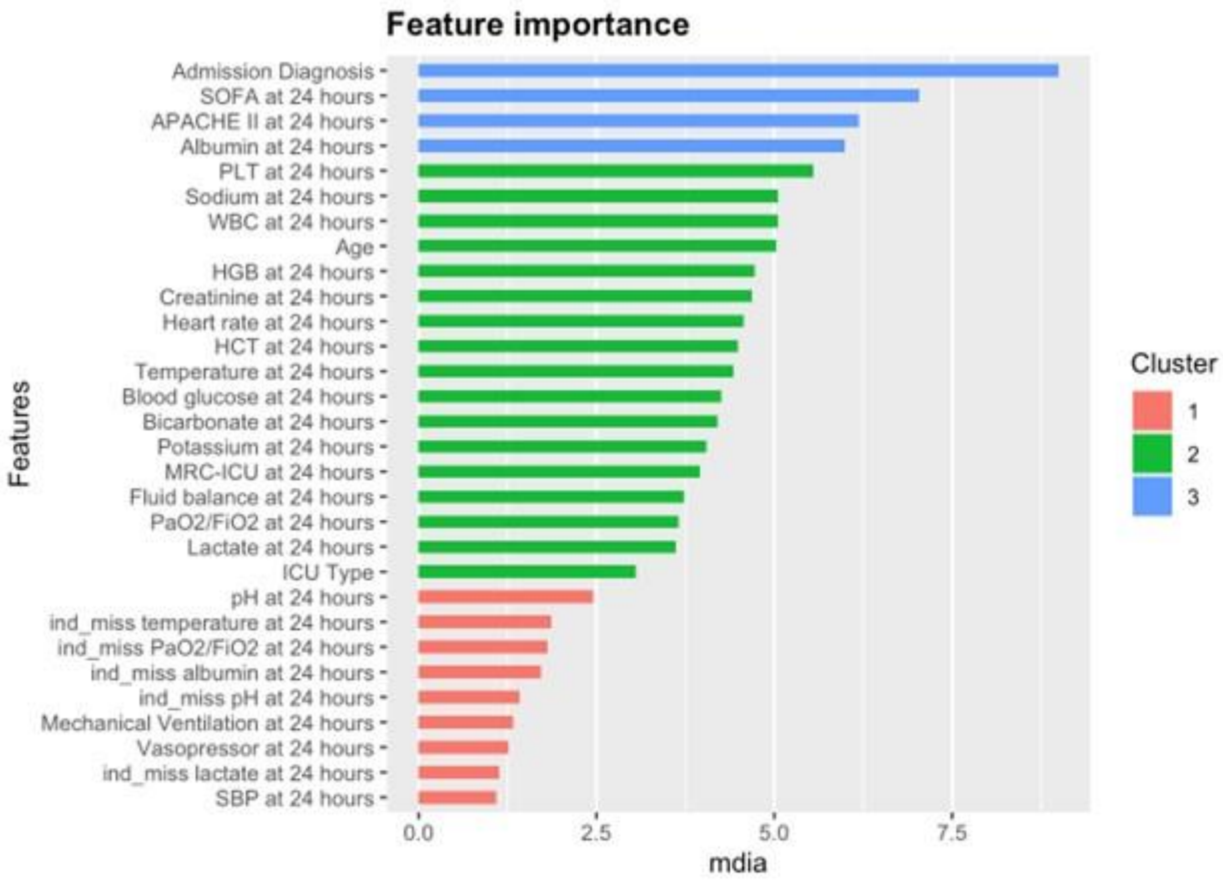

Standard severity of illness metrics (APACHE II, SOFA) and admission diagnosis demonstrated high feature importance in Random Forest ML-based models for mortality prediction in critically ill adult patients admitted to the ICU. Medication data as summarized in the MRC-ICU score demonstrated moderate feature importance similar to factors considered to have high clinical value in patient assessment and outcomes prediction (e.g., fluid balance, lactate, PaO2/FiO2 ratio). ML-based modeling is capable of identifying and characterizing complex relationships that may not be evident using traditional modeling techniques.

Supplemental Figure 2. Feature importance graph for SVM on test set

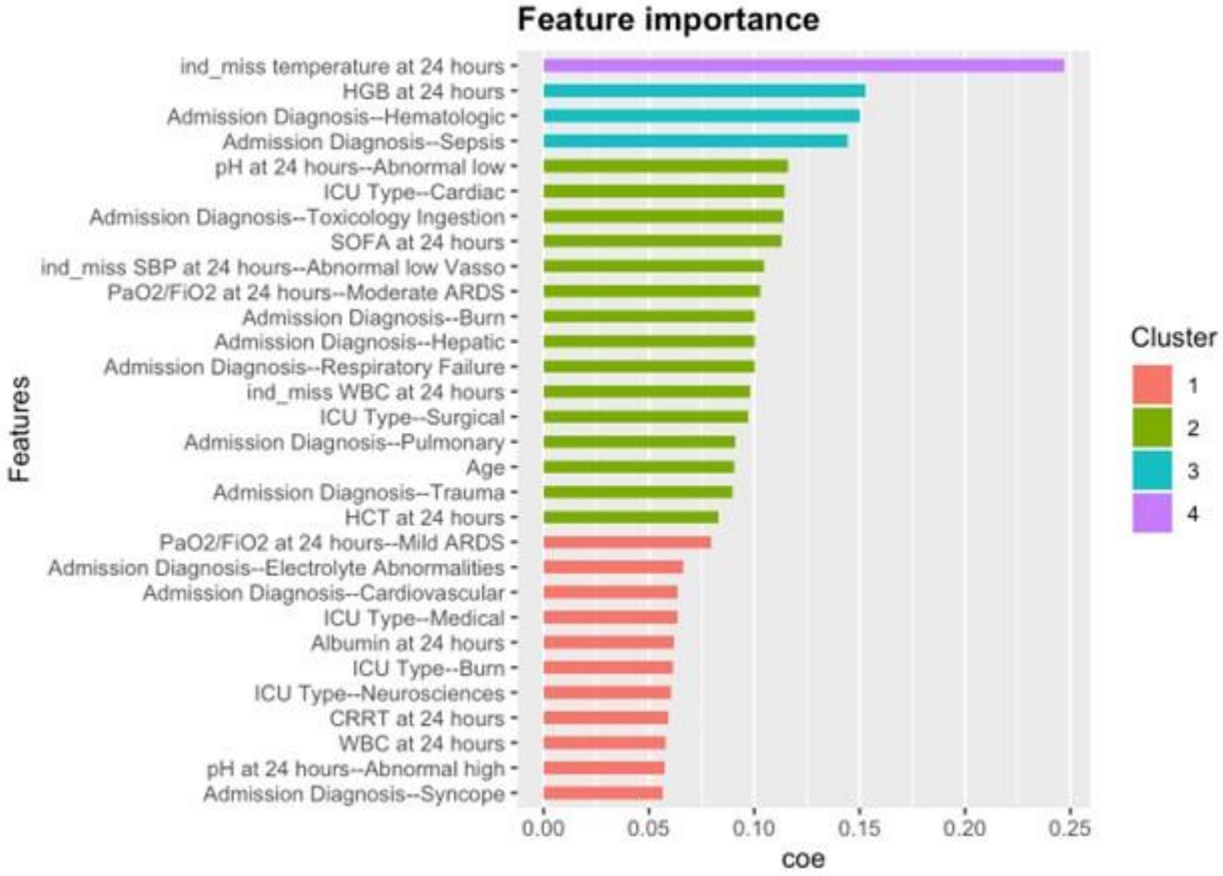

SVM ML-based models for mortality prediction in critically ill adult patients admitted to the ICU identified unique variables with high feature importance, including a indicator of missingness for temperature. One of the notable pitfalls of ML-based models is the "black box" nature of their output.

Supplemental Figure 3. AUROCs for hospital mortality prediction on UNC 5000 validation set

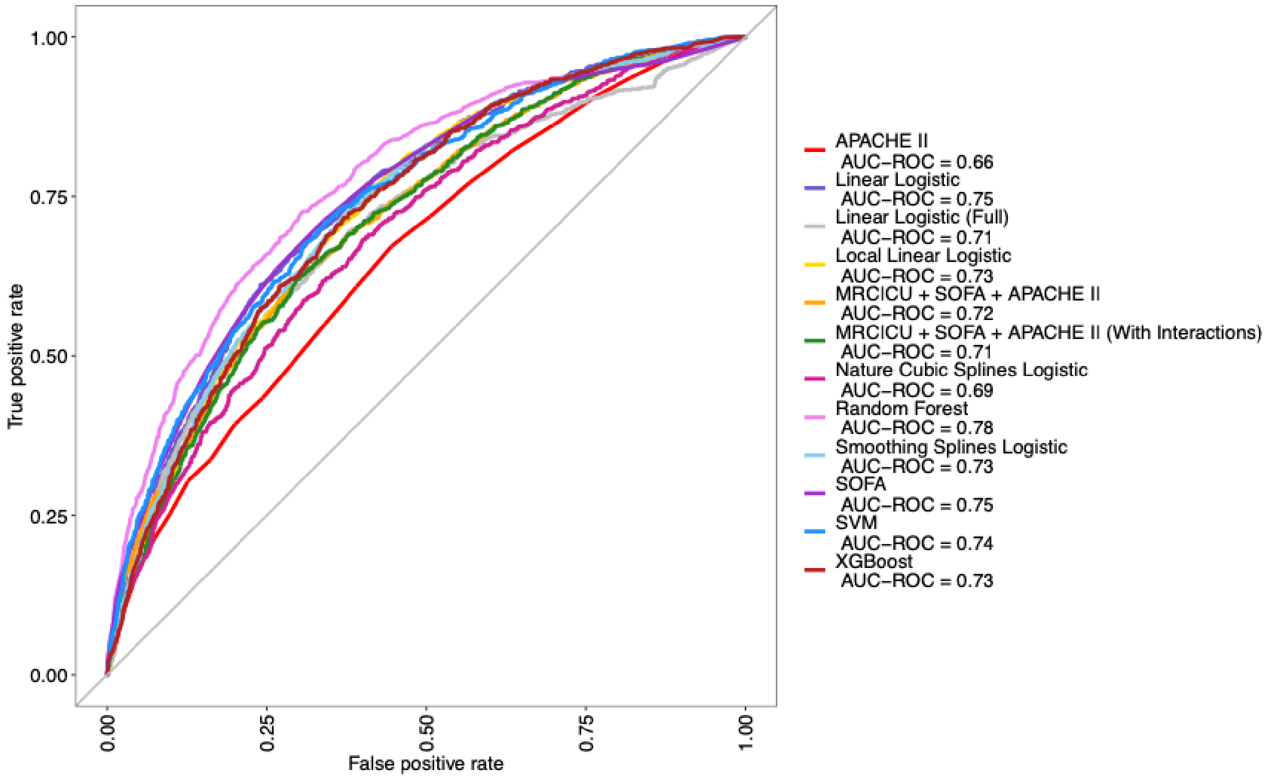

Performance of all models was lower on the UNC 5000 validation set than on the test set. Random Forest demonstrated the most consistent performance from test set to validation set.

Supplemental Figure 4. AUROCs for hospital mortality prediction on OHSU validation set

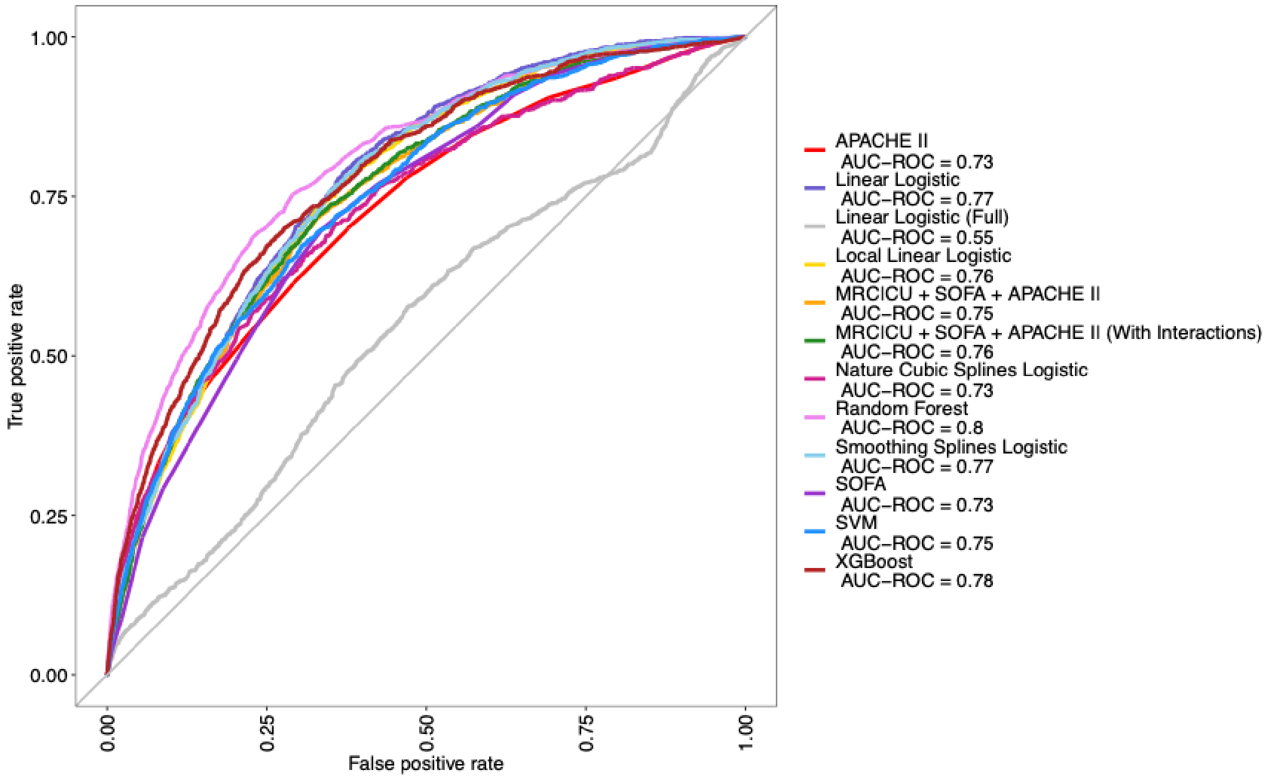

Performance of all models was lower on the OHSU validation set than on the test set. Random Forest demonstrated the most consistent performance from test set to validation set.

Supplemental Figure 5. Calibration curves of models for hospital mortality prediction on testing set

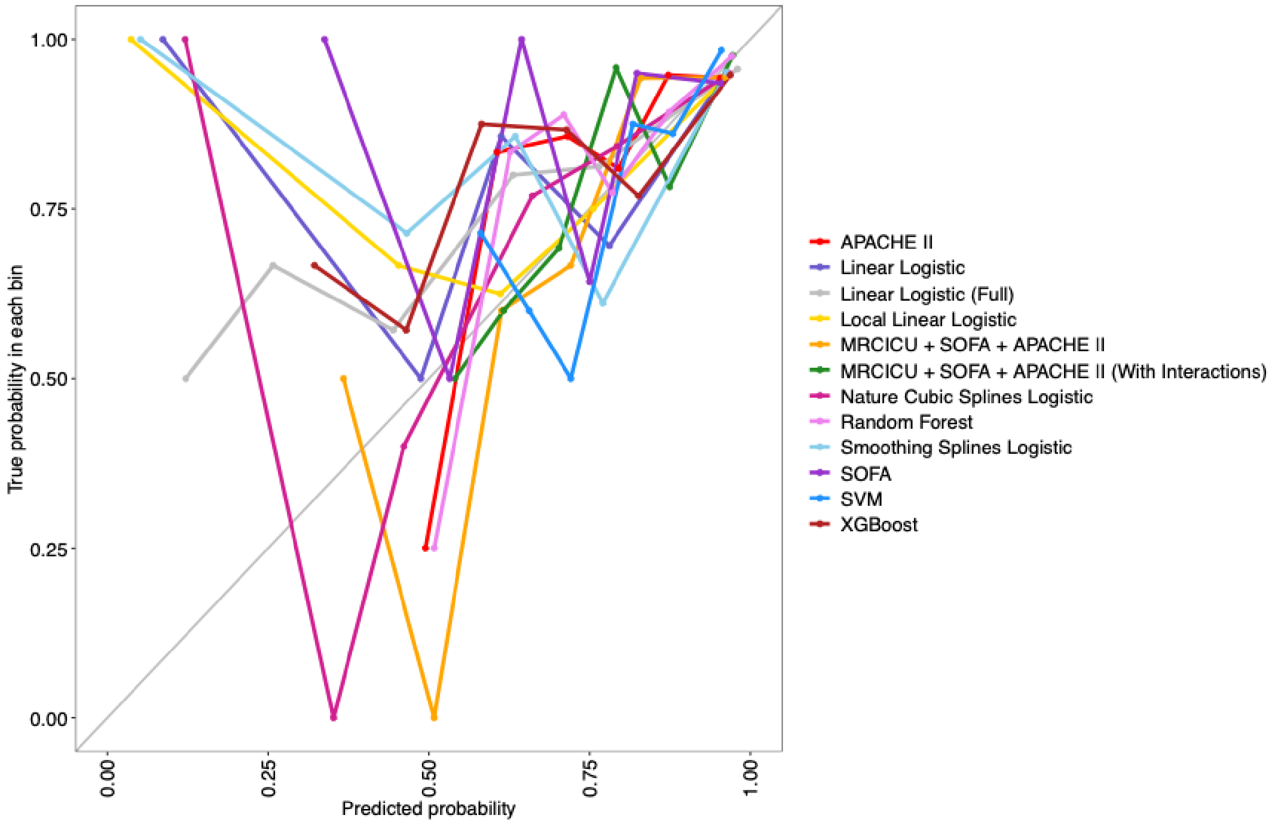

Supplemental Figure 6. Calibration curves of models for hospital mortality prediction on UNC 5000 validation set

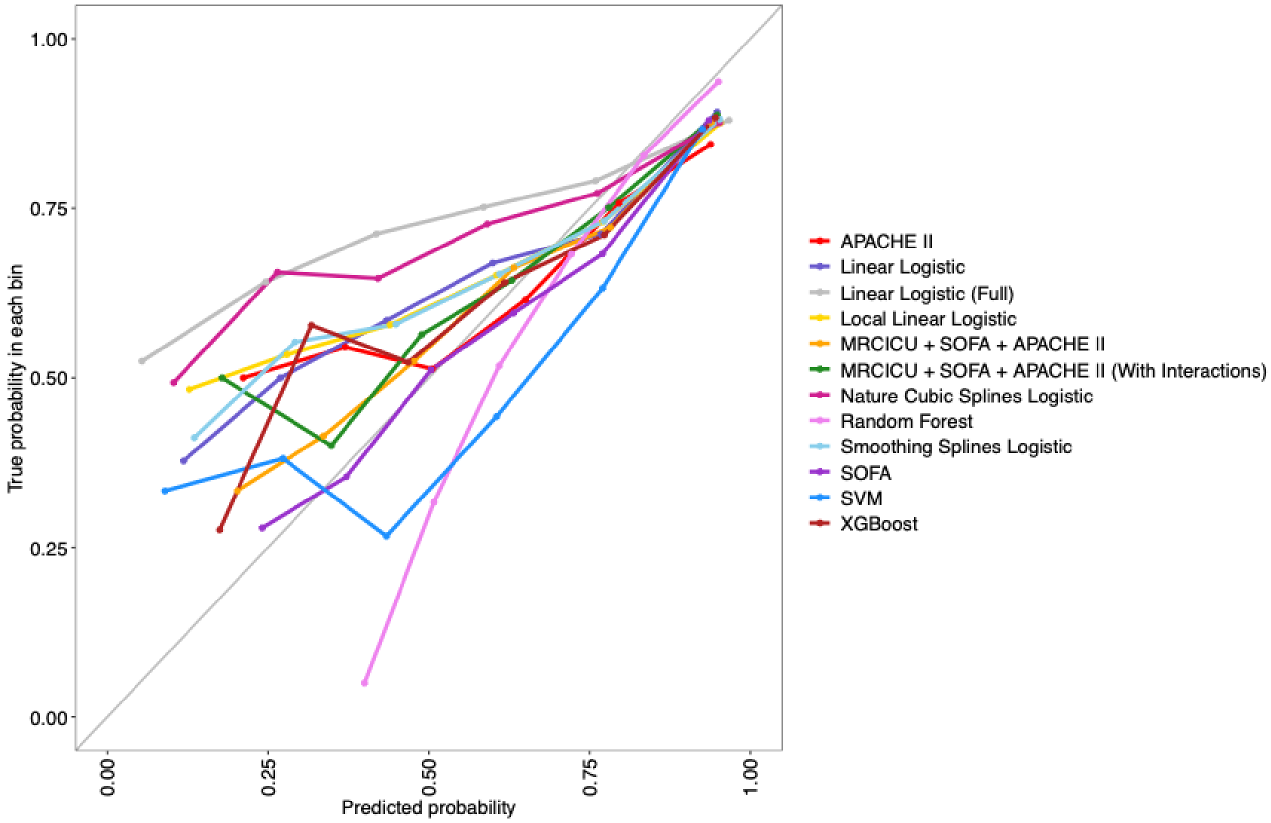

Supplemental Figure 7. Calibration curves of models for hospital mortality prediction on OHSU validation set

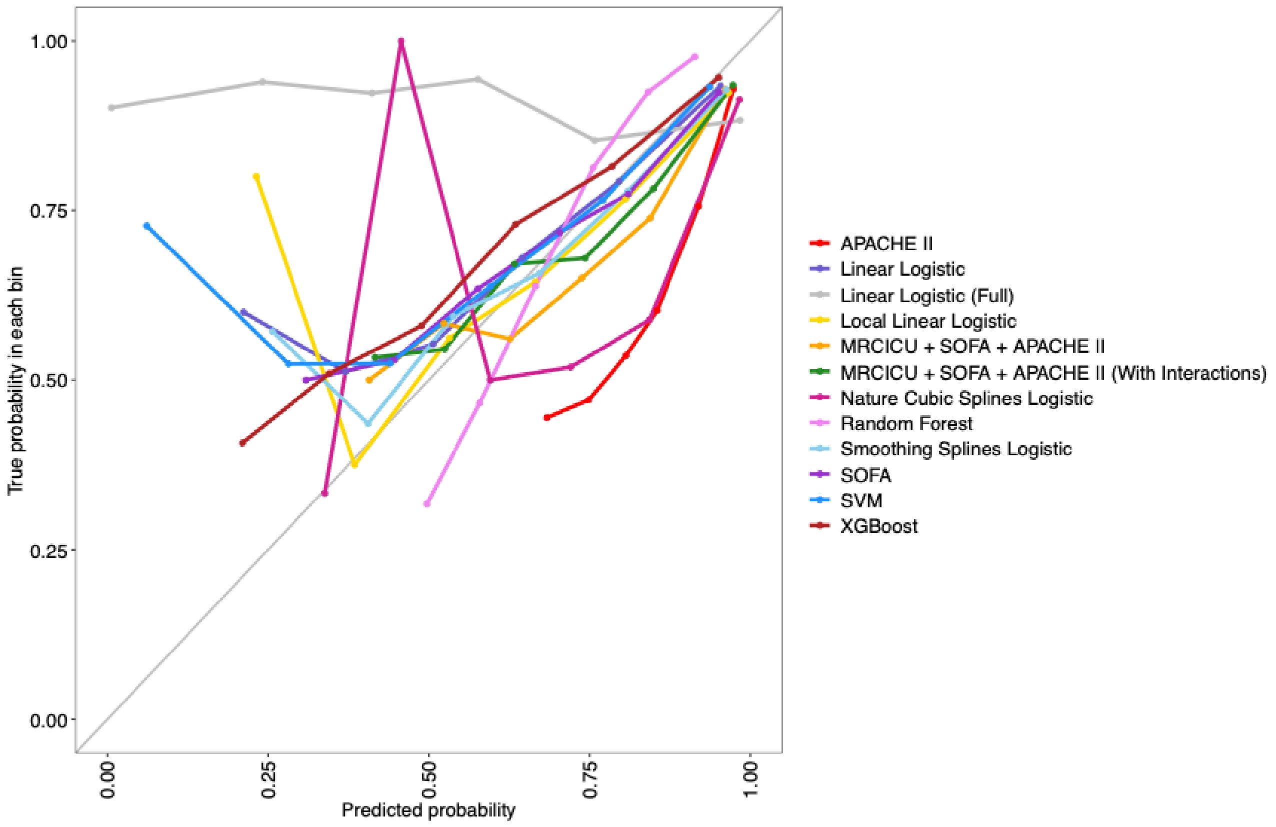

Supplement: Supplementary file 1 [file cc9-7-e1331-s001.pdf]
